# Sleep spindle detection based on non-experts: A validation study

**DOI:** 10.1371/journal.pone.0177437

**Published:** 2017-05-11

**Authors:** Rui Zhao, Jinbo Sun, Xinxin Zhang, Huanju Wu, Peng Liu, Xuejuan Yang, Wei Qin

**Affiliations:** Sleep and Neuroimage Group, School of Life Sciences and Technology, Xidian University, Xi’an, Shaanxi, China; Boston Children's Hospital / Harvard Medical School, UNITED STATES

## Abstract

Accurate and efficient detection of sleep spindles is a methodological challenge. The present study describes a method of using non-experts for manual detection of sleep spindles. We recruited five experts and 168 non-experts to manually identify spindles in stage N2 and stage N3 sleep data using a MATLAB interface. Scorers classified each spindle into definite and indefinite spindle (with weights of 1 and 0.5, respectively). First, a method of optimizing the thresholds of the expert/non-expert group consensus according to the results of experts and non-experts themselves is described. Using this method, we established expert and non-expert group standards from expert and non-expert scorers, respectively, and evaluated the performance of the non-expert group standards by compared with the expert group standard (termed EGS). The results indicated that the highest performance was the non-expert group standard when definite spindles were only considered (termed nEGS-1; F1 score = 0.78 for N2; 0.68 for N3). Second, four automatic spindle detection methods were compared with the EGS. We found that the performance of nEGS-1 versus EGS was higher than that of the four automated methods. Our results also showed positive correlation between the mean F1 score of individual expert in EGS and the F1 score of nEGS-1 versus EGS across 30 segments of stage N2 data (r = 0.61, P < 0.001). Further, we found that six and nine non-experts were needed to manually identify spindles in stages N2 and N3, respectively, while maintaining acceptable performance of nEGS-1 versus EGS (F1 score = 0.79 for N2; 0.64 for N3). In conclusion, this study establishes a detailed process for detection of sleep spindles by non-experts in a crowdsourcing scheme.

## Introduction

Sleep spindles are characterized by a waxing and waning shape, within the sigma frequency range (11~16 Hz) with a minimum duration of 0.5s, and they are a key hallmark of stage 2 (N2) non-rapid eye movement (NREM) sleep [1]. The roles of sleep spindles in basic and clinical sleep research are well documented recently. Much evidence indicates that the functional significance of sleep spindles has been implicated in intelligence [2, 3] and sleep-dependent memory consolidation [4, 5], which suggests that sleep spindles may be considered both as a physiological index of intellectual abilities and a marker of the capacity for learning [6, 7]. Clinically, alterations of sleep spindle characteristics are obtained in many diseases, such as Parkinson’s [8, 9], schizophrenia [10, 11], autism [12], insomnia [13, 14] and Alzheimer’s disease [15], which may represent a biomarker of illness and have important supplementary diagnostic value. Therefore, it is imperative to accurately detect spindles.

Although visual inspection by experts is the gold standard of sleep spindle detection, with the rapid increase in research on sleep spindles, various automated methods of spindle detection have been proposed to reduce subjective biases and increase reliability and objectivity. The major advantages of automated methods are faster, more reproducible and systematic scoring. They extract features from EEG data and apply specific thresholds to identify features corresponding to sleep spindles. A standardized band-pass filter or custom frequency range filter and amplitude-threshold approach has been commonly used in research literature and reported approximately 90% sensitivity [16–22]. Time-frequency analysis method also has been applied in spindle detection with wavelet transformation and matching pursuit [11, 23, 24]. More recently, sophisticated automatic sleep spindle detection methods using artificial neural networks have been developed and reported high agreement with experts (ranging from 85% to 96%) [8, 25–27]. Although there is ample evidence that many automated methods have an acceptable agreement with experts, they have the limitations that they are known for occasional problems with differentiating ambiguous oscillation signals (e.g., alpha versus spindles). They can also be highly influenced by the algorithm settings chosen by researchers (e.g., spindle duration, frequency, and amplitude characteristics) [28–31].

To make up for the time-consumingness of manually detection spindles by experts and the limitations of automated methods, crowdsourcing by non-experts may be another potential technique. It divides a large, complex task into smaller parts which are allocated to a group of untrained individuals in the general public [32]. Crowdsourcing has been used successfully to decipher complex protein folding structures with an online game (http://foldit.wikia.com/wiki/Foldit_Wiki) and solve complex medical cases (https://www.crowdmed.com/). In addition, it has been used in video-based assessment of surgical technique with inexperienced users who perform as well as experts [33]. Early studies have also shown that this technique has the potential to significantly advance medical image analysis delivery which can provide a combination of accuracy and cost effectiveness [34, 35]. These studies suggest that crowdsourcing may be a significant potential technique in many fields. Remarkably, Warby et al. [36] in Nature Methods investigated the agreement in spindle detection between expert scorers, non-experts based on crowdsourcing spindle identification, and automatic detection algorithms. They found that concordance was the strongest among experts, followed by non-experts, and weakest among automated methods. This work opens an attractive avenue for detecting sleep spindles in a crowdsourcing scheme.

Expert visual identification of sleep spindles is based on years of experience. Non-experts can copy or imitate experts’ manual spindle inspection and flexibly distinguish spindles among varying EEG background activity, suggesting that crowdsourcing by non-experts may be a promising method of spindle detection. If we apply this method practically, sleep spindle detection should be only based on the judgment of non-experts (i.e., independent of that of experts). However, the study by Warby et al. didn’t satisfy this condition. The goal of the present study is to evaluate spindle identification performance by a group of non-experts which is independent of the judgment of experts, in a crowdsourcing scheme.

In the present study, we crowdsourced spindle identification of stage N2 and stage N3 sleep EEG data to 168 non-expert scorers using a MATLAB interface. To generate the non-expert group standard, we optimized the thresholds of the non-expert group consensus with reference to non-experts themselves rather than the constant threshold of Warby’s study. To evaluate performance, we compared the non-expert group standard with the expert group standard (EGS) which was generated from five experts. These experts manually scored sleep spindles in all EEG data sets using the same interface. To compare with the non-expert group standard, we also implemented four automated spindle methods commonly used in research literature. If the non-expert group standard met the accuracy requirement, we then investigated its efficiency by determining the smallest number of non-experts required to identify spindles. Overall, we found that when definite spindles were only considered, the non-expert group standard (termed nEGS-1) showed acceptable agreement with the EGS. The agreement of nEGS-1 with EGS was better than that of the automatic detection methods we tested. We also found that for stage N2 and N3 sleep data at least six and nine non-experts are needed to maintain the acceptable performance of nEGS-1 versus EGS, respectively.

## Materials and methods

### EEG data set

We anonymously chose the EEG data set from 30 healthy, young subjects (mean age, 22.74 ± 1.24 years; 11 females, 19 males), and randomly selected 10 minutes of artifact-free N2 sleep data and 10 minutes of artifact-free N3 sleep data from each subject. Each data segment was chosen in one continuous chunk which was randomly selected from the first half of a night. In total, 60 segments of sleep data constituted the EEG data set (30 segments of N2 sleep data and 30 segments of N3 sleep data) with a sampling rate of 500 Hz. All subjects displayed regular sleeping patterns, had no sleep disorders, and were free of any drugs that would disrupt sleep architecture. All procedures performed in this study involving human participants were approved by the institutional research ethics committee of the Xijing Hospital of the Fourth Military Medical University. All subjects gave written informed consent for their participation, data collection and usage.

### Spindle identification interface

To visually identify sleep spindles, we developed a spindle identification interface based on MATLAB. The interface displayed 10 seconds of band-pass-filtered (0.3 ~ 35 Hz) EEG data from the C3-M2 channel at a time. The presented voltages of vertical coordinates ranged from -75 to 75μV, and the vertical dashed lines marked 1s intervals. Scorers drew a line around a sleep spindle, and pressed a Sure button (definite spindle) or a Not Sure button (indefinite spindle). The start and end of the line represented the start and end of the spindle exactly, and the length was the duration of the spindle. We weighted definite spindles, indefinite spindles and the non-spindle parts with values of 1, 0.5 and 0, respectively. In addition, scorers could delete marked spindles, and change a score from definite spindle to indefinite spindle and vice versa. For each data segment, a vector consisting of 1, 0.5 and 0 was generated which was to be used for further analysis.

### Experimental protocol

A manual on how to identify spindles was written by a technologist who had scored sleep stages for many years. The manual discussed the methods of spindle identification based on the spindle’s shape, frequency, duration and amplitude, and gave various spindle examples to teach scorers (experts and non-experts) how to correctly and accurately identify spindles. Sleep spindles stood out as being distinct from the surrounding EEG signal with a waxing and waning shape. They oscillated at 11–16 cycles per second which could be determined by counting the number of wave peaks per second. Spindles were at least 0.5 seconds in duration according to the American Academy of Sleep Medicine [1]. For the spindle amplitude, there is no strict criterion, but it is a little larger than the waves around it. All scorers were instructed to read this manual and performed a training session before the actual spindle identification task.

We recruited another five technologists who had scored sleep stages for at least three years as our experts to identify spindles in all EEG data. These experts were recruited by word of mouth. We sent a package containing the spindle identification interface, the operating instructions for the interface, and all 60 segments of EEG data to the experts by email. Experts were instructed to read the manual on spindle identification and performed a training session before the actual spindle identification task. The manual writer asked every expert if he/she had read the manual and graded the performance of the training session. Not until their performance exceeded 70 did they participate in the final experiment. The average score of experts was 86.76 ± 5.12. The collection of the expert data took approximately 1 month. Finally, we provided some rewards for their work.

Posting on a forum and sending a small advertisement were used to recruit 168 non-experts who were not familiar with sleep spindles. The forum was a university forum and its members were mainly students of Xidian University. The advertisement was self-made by writing the recruitment information. The non-experts were undergraduate and master students of Xidian University, other than students of our team. They specialized in computer science, electronic engineering or communication engineering, and all of them were not familiar with sleep spindles. All non-experts were instructed to study the same manual on how to identify spindles and performed the same training session as experts. Similarly, the manual writer asked every non-expert if he/she had read the manual and graded the performance of training session. Not until their performance exceeded 60 did they participate in the final experiment. The average score of non-experts was 69.84 ± 7.24.

Non-experts performed the actual spindle identification task in a laboratory that collects behavioral data. A maximum of five non-experts identified spindles independently at a time. Everyone identified six segments of EEG data, including three segments of stage N2 and three segments of stage N3 data. These six data segments were randomly extracted from the 60 segments of the EEG data set and were not derived from the same subject. Each data segment was identified by at least 20 non-experts. There was no limit on how many data segments each individual non-expert should score from the dataset, but the same non-expert was permitted to score the same data segment only once. Non-experts were paid according to their finished workload. The average time spent on their work was 108 ± 36 min, and the collection of the non-expert data took approximately 18 days. No experts or non-experts opted out of this study once it began. Here, we declared that all authors have no contact with any spindle scorers or the sleeping participants.

### Data analysis

**Fig 1:**
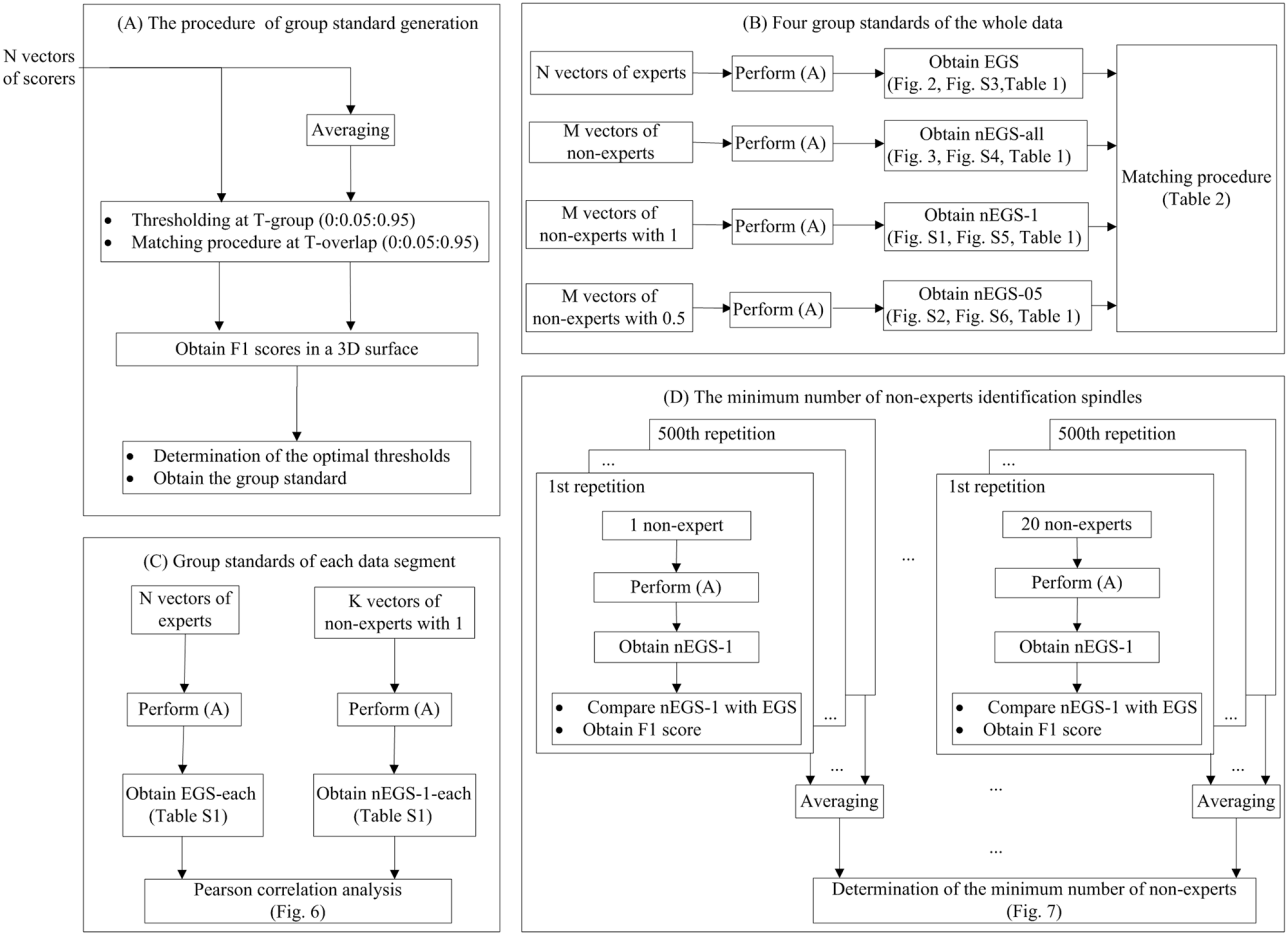
Schematic diagram of the analysis pipeline. The pipeline is comprised by four blocks. In the first block (A), the method of generating group standard is elaborated. N vectors are averaged to obtain the group consensus. A group vector and N individual vectors were found by applying a group threshold (T-group = 0:0.05:0.95) to the group consensus and the N vectors. Using the matching procedure, the N individual vectors were compared with the group vector at varying overlap thresholds (T-overlap = 0:0.05:0.95) to obtain the F1 score. The mean F1 scores corresponding to varying T-group and T-overlap values were calculated by averaging the F1 scores of N scores. Finally, the optimal T-group was determined from the 3D surface by maximizing the mean F1 score of all individuals in the group, and the best T-overlap was selected by finding the first point at which the difference between the two adjacent values of the mean F1 score at the best T-group was more than 0.002. The group standard was then established from the group consensus at the optimal T-group. The second block (B) illustrates the establishment of four group standards (EGS, nEGS-all, nEGS-1 and nEGS-05) by performing the first block. The EGS (expert group standard) was generated from five experts. The nEGS-all (non-expert group standard with all spindles) was established from 168 non-experts when both definite spindles and indefinite spindles were considered. The nEGS-1 (non-expert group standard with definite spindles) was obtained from 168 non-experts when definite spindles were only considered. The nEGS-05 (non-expert group standard with indefinite spindles) was obtained from 168 non-experts when indefinite spindles were only considered. Then, the three non-expert group standards (nEGS-all, nEGS-1 and nEGS-05) are compared with EGS using a matching procedure. The third block (C) illustrates the establishment of group standards for each data segment (EGS-each and nEGS-1-each) by performing the first block. For each data segment (n = 30), the EGS-each (expert group standard of each data segment) was obtained from five experts. The nEGS-1-each (non-expert group standard of each data segment only containing the definite spindles) was established from 168 non-experts. Then, Pearson correlation analysis is performed between the performance of EGS-each and nEGS-1-each across 30 data segments. The fourth block (D) determines the minimum number of non-experts required to identify spindles. For each data segment, *n* (*n* = 1,2,3,…,20) non-experts identified the data. We generated the nEGS-1 from these *n* non-experts by performing the first block, and obtained the F1 score of nEGS-1 versus EGS using the matching procedure. This approach was repeated 500 times. The mean F1 score of nEGS-1 versus EGS was calculated across 500 repetitions. Then, we determined the minimum number of non-experts by finding the first point at which the mean F1 score of nEGS-1 versus EGS approached a stable value.

A schematic diagram of the analysis pipeline was illustrated in Fig 1. The pipeline was comprised by four blocks. The procedures of these four blocks were elaborated in the following section.

#### The procedure of group standard generation

The generation of group standard was based on the study of Warby et al. [36] (Fig 1A). We took EGS (see definitions in S4 Table) in stage N2 sleep data as an example to provide a brief outline. The generation of EGS was performed on 150 vectors (30 segments of data multiplied by 5 experts), each of which was the result of one expert’s identification of one data segment. This vector contained 300,000 points (500 Hz × 600 seconds) comprising 10 minutes of data and composed of the values 1, 0.5 and 0 (1 = definite spindle, 0.5 = indefinite spindle, 0 = no spindle part).

This procedure included three stages. The first stage included the following three operations for each data segment. 1) The expert group consensus was obtained by averaging the vectors of the five experts at each sample point. 2) The expert group vector and five binary individual expert vectors were found by applying a group threshold (T-group, which increased from 0 to 0.95 in increments of 0.05) to the expert group consensus and the five individual expert scores, respectively. 3) The five individual expert vectors were compared with the expert group vector at varying overlap thresholds (T-overlap, which increased from 0 to 0.95 in increments of 0.05) using a matching procedure (see the methods in S1 File), and a classic 2 × 2 contingency table of true positive (TP), false positive (FP) and false negative (FN) was obtained.

The matching procedure was used to classify two spindles, which were respectively identified in the individual expert vectors and expert group vector, into the contingency table. Specifically, if the overlap ratio (the intersection/union score, O_ED_) exceeded T-overlap, these two spindles were classified as TP; otherwise, the spindle in the binary individual expert vector was classified as FP, and the spindle in the expert group vector was classified as FN. Detailed descriptions and several special situations of the matching procedure are shown in S1 File.

$${\text{O}}_{\text{ED}}\;=\;\frac{\text{E }\cap \text{ D}}{\text{E }\cup \text{ D}}$$ (1)

$$\text{D }=\{\begin{array}{ll} \text{TP} & {\text{O}}_{\text{ED}}\;>\text{ T-overlap}\\ \text{FP} & {\text{O}}_{\text{ED}}\;\leq \text{ T-overlap} \end{array}$$ (2)

$$\text{E }=\{\begin{array}{ll} \text{TP} & {\text{O}}_{\text{ED}}\;>\text{ T-overlap}\\ \text{FN} & {\text{O}}_{\text{ED}}\;\leq \text{ T-overlap} \end{array}$$ (3)

Here, E was one spindle in the expert group vector. D was one spindle in the individual expert vector.

Therefore, we obtained the TP, FP and FN values which respectively corresponded to various data segments (n = 30), T-groups (n = 20), T-overlaps (n = 20) and experts (n = 5). In total, 60,000 (30 × 20 × 20 × 5) series of [TP, FP, FN] were generated over all cases.

In the second stage, the TP, FP and FN values of each expert across 30 segments of data were added. The recall, precision and F1 scores (20 × 20 × 5 cases), which all ranged from 0–1, were calculated. Then, the mean F1 score was calculated by averaging the five experts’ F1 scores. Finally, a 20 × 20 matrix of mean F1 scores was obtained for varying T-group and T-overlap values and shown on a three-dimensional (3D) surface (X-axis: T-group, Y-axis: T-overlap, Z-axis: mean F1 score).

$$\text{recall }=\;\frac{\text{TP}}{\text{TP }+\text{ FN}}$$ (4)

$$\text{precision }=\;\frac{\text{TP}}{\text{TP }+\text{ FP}}$$ (5)

$$\text{F1 score }=\;\frac{2\;\times \text{ recall }\times \text{ precision}}{\text{recall }+\text{ precision}}$$ (6)

In the third stage, we determined the best T-group and T-overlap values from the 3D surface. The best T-group was selected by maximizing the mean F1 score, and the expert group consensus at the best T-group was the EGS which was then used as the gold standard. The best T-overlap specified the required overlap rate to classify TP spindles. However, the T-overlap corresponding to the maximal mean F1 score was 0, which was too relaxed for evaluation of group standard performance. Therefore, after determining the optimal T-group, we analyzed the mean F1 scores of the best T-group at varying T-overlap values and found the first point at which the difference between the two adjacent mean F1 scores was greater than 0.002. The overlap threshold corresponding to this point was identified as the best T-overlap value. The mean F1 score at this point was the mean performance of all individual experts in the EGS. Finally, we established the EGS and obtained three parameters: the best T-group, the best T-overlap and the mean F1 score (termed $\bar{\text{F1 score}}$). For further details, see Warby et al. [36].

In this section, we described a method of generating the group standard, taking the EGS for example. Briefly, using the matching procedure, individual experts’ scores were compared with the expert group consensus at varying T-group and T-overlap values, and the classic contingency table of TP, FP and FN was obtained. Then, the mean F1 score of all experts was calculated from the contingency table at varying T-groups and T-overlaps. Finally, the optimal thresholds were determined from the 3D surface. The expert group consensus at the best T-group was nominated as the EGS (Fig 1A).

### Four group standards of the whole data

The four group standards were the EGS, the non-expert group standard with all spindles (both definite spindles and indefinite spindles were considered, denoted as nEGS-all), the non-expert group standard with definite spindles (definite spindles were only considered, denoted as nEGS-1) and the non-expert group standard with indefinite spindles (indefinite spindles were only considered, denoted as nEGS-05). The three non-expert group standards (nEGS-all, nEGS-1 and nEGS-05) were established from 168 non-experts using the similar procedure as the EGS (Fig 1B). Detailed descriptions of the generation of the three non-expert group standards are shown in the methods of S1 File. In this procedure, the optimal T-group value was determined by maximizing the mean F1 score of all individuals in the non-expert group (rather than maximizing the F1 score generated by compared the non-expert group consensus with the EGS [36, 37]). Each group standard had its own optimal T-overlap instead of a constant threshold [36]; thus, the non-expert group standard established in our procedure is generated by using data only from non-experts themselves (i.e., independent of experts).

The four group standards of stages N2 and N3 data were obtained. Table 1 shows several parameters that were collected for each group standard, including the number of experts/non-experts in each standard, the best T-group, T-overlap, $\bar{\text{F1 score}}$ (the mean F1 score of the group standard corresponding to the optimal thresholds), and the range of F1 scores across all experts or non-experts in each standard.

**Table 1:** Parameters of four group standards in stage N2 and N3 sleep data.

Stage	Standard	Number	T-group	T-overlap	$\bar{\text{F1 score}}$	F1 score range	$\bar{\text{F1 score-leave}}$
**N2**	**EGS**	5	0.30	0.45	0.84 ± 0.02	0.82 ~ 0.88	0.79 ± 0.03
	**nEGS-all**	168	0.35	0.30	0.72 ± 0.10	0.24 ~ 0.88	0.70 ± 0.10
	**nEGS-1**	168	0.35	0.30	0.68 ± 0.11	0.24 ~ 0.90	0.66 ± 0.11
	**nEGS-05**	168	0.05	0.30	0.27 ± 0.13	0.003 ~ 0.64	0.17 ± 0.08
**N3**	**EGS**	5	0.30	0.40	0.74 ± 0.05	0.66 ~ 0.79	0.63 ± 0.06
	**nEGS-all**	168	0.30	0.30	0.59 ± 0.12	0.22 ~ 0.84	0.55 ± 0.12
	**nEGS-1**	168	0.35	0.25	0.53 ± 0.14	0.15 ~ 0.79	0.47 ± 0.13
	**nEGS-05**	168	0.05	0.35	0.26 ± 0.13	0.027 ~ 0.56	0.18 ± 0.09

Data are presented as mean ± standard deviation unless otherwise indicated. The EGS was the expert group standard. The nEGS-all was the non-expert group standard with all spindles when both the weighted value of 1 and 0.5 were considered. The nEGS-1 was the non-expert group standard with definite spindles when the weighted value of 1 was only considered. The nEGS-05 was the non-expert group standard with indefinite spindles when the weighted value of 0.5 was only considered. The Number was the number of experts/non-experts in each standard. The T-group was the best group threshold. The T-overlap was the best overlap threshold. The $\bar{\text{F1 score}}$ was the mean F1 score of individuals in a group related to its own group at the best T-group and T-overlap. The F1 score range was the range of F1 scores of 168 non-experts or five experts. The $\bar{\text{F1 score-leave}}$ was the mean F1 score of all experts or non-experts in the leave-one-out analysis. All standards were established by thresholding the group consensus at the T-group.

To evaluate which non-expert group standard was the best, we compared the three non-expert group standards with the EGS at their respective optimal thresholds using the matching procedure, and obtained the TP, FP, FN, recall, precision and VS-F1-score values (Table 2). The VS-F1-score was the F1 score of one standard compared with the EGS using the matching procedure (see methods in S1 File), representing the similarity between one standard and the gold standard.

**Table 2:** The performance of the three non-expert group standards and four automated methods compared to the expert group standard in stage N2 and N3 sleep data.

Stage	Standard	Number	TP	FP	FN	Recall	Precision	VS-F1-score
**N2**	**nEGS-all**	168	543	339	12	0.98	0.62	0.76
	**nEGS-1**	168	529	265	26	0.95	0.67	0.78
	**nEGS-05**	168	151(138)	303(84)	404	0.27	0.33	0.30
	**AS1**	N/A	472	508	83	0.85	0.48	0.62
	**AS2**	N/A	386	267	169	0.70	0.59	0.64
	**AS3**	N/A	179	78	376	0.32	0.70	0.44
	**AS4**	N/A	426	2458	129	0.77	0.15	0.25
**N3**	**nEGS-all**	168	149	221	3	0.98	0.40	0.57
	**nEGS-1**	168	135	113	17	0.89	0.54	0.68
	**nEGS-05**	168	28(23)	124(29)	144	0.18	0.16	0.17
	**AS1**	N/A	143	775	9	0.94	0.16	0.27
	**AS2**	N/A	127	448	25	0.84	0.22	0.35
	**AS3**	N/A	46	47	106	0.30	0.49	0.38
	**AS4**	N/A	125	2848	27	0.82	0.04	0.08

The nEGS-all was the non-expert group standard with all spindles when both the weighted values of 1 and 0.5 were considered. The nEGS-1 was the non-expert group standard with definite spindles when the weighted value of 1 was only considered. The nEGS-05 was the non-expert group standard with indefinite spindles when the weighted value of 0.5 was only considered. The AS1, AS2, AS3 and AS4 were the automatic standards obtained by the first, second, third and fourth automated method, respectively. The Number was the number of non-experts in each standard. The TP is true positive. The FP is false positive. The FN is false negative. TP divided by the sum of TP and FN equals to the Recall. TP divided by the sum of TP and FP equals to the Precision. The VS-F1-score is the F1 score of each group standard compared with the expert group standard. Brackets denote the number of the same TP or FP spindles of nEGS-05 and nEGS-1.

Furthermore, we implemented four automated spindle detection methods that are commonly used in research literature. For the first automated method [38, 39], each data segment was band-pass filtered from 11 to 16 Hz, using a linear phase finite impulse response filter. The root mean square of the band-pass filtered signal was calculated with a time resolution of 250 milliseconds using a time window of 250 milliseconds. A spindle was detected if the root mean square exceeded its 95^th^ percentile for 0.5 seconds.

The second method was a wavelet-based algorithm [11, 24]. Each data segment was performed the wavelet transformation using a complex Morlet wavelet, and then the moving average of the wavelet scale was calculated (a 100 milliseconds sliding window). Spindles were detected where the wavelet signal exceeded a threshold (4.5 times the moving average of the wavelet scale) for a minimum of 0.5 s.

We also implemented an automated method using spindle envelope to determine the start and the end of a spindle [10, 40]. Each data segment was band-pass filtered between 11 and 16 Hz, and the envelope was calculated by using the local maxima of the rectified signal. Then the lower and upper thresholds for spindle detection were determined as two and eight times the mean amplitude of signal, respectively. Spindles occurred when the mean amplitude of signal exceeded the upper threshold. The start and the end of the spindle were determined when the mean amplitude of signal was below the lower threshold.

Finally, the fourth automated method calculated spindle frequency boundaries and spindle detection amplitude criteria of slow and fast spindles using the average amplitude spectrum during N2 sleep [18, 21]. Then each data segment was band-pass filtered in slow and fast sleep spindle frequency boundaries. Slow spindles or fast spindles were detected when the amplitude criteria were exceeded.

For each automated method, we assigned a weight of 1 to detected spindle or 0 to no spindle part and generated the automatic standards (termed AS1, AS2, AS3 and AS4, respectively). Then, we compared the automatic standards with the EGS at varying overlap thresholds (T-overlap = 0:0.05:0.95) using the matching procedure. The best overlap threshold was determined using the above method (i.e., by selecting the first point at which the difference between the two adjacent F1 scores was more than 0.002). The F1 score of this point was called the VS-F1-score. The TP, FP, FN, recall, precision and VS-F1-score values of each automatic standard versus EGS at the best overlap threshold are listed in Table 2.

#### Group standards of each data segment

Electric potentials’ amplitudes are affected by the skull thickness: a thicker skull results in a greater electrical resistance, which reduces surface EEG amplitudes according to Ohm’s law [41, 42]. When the EEG amplitudes are reduced, the frequency (determined by counting the number of wave peaks per second) is difficult to calculate, as there are more low-amplitude burrs that are easily confused with wave peaks, and spindle amplitude is not significantly larger than that of the surrounding waves. Scorers might miss or be in doubt about such spindle. Therefore, the reduced EEG amplitudes would enhance the difficulty of spindle identification, suggesting that data’s attributes influence human spindle identification performance. To examine this effect, for each data segment, we independently established the expert group standard (EGS-each) from the expert scorers and the non-expert group standard with definite spindles (nEGS-1-each) from the non-expert scorers who identified this data (Fig 1C). Detailed descriptions of the generation of EGS-each and nEGS-1-each are shown in the methods of S1 File.

For each data segment, we obtained the best T-group, T-overlap, and $\bar{\text{F1-score-each}}$ (the specific definition see below) of EGS-each and nEGS-1-each. The means of these parameters (mean_30_T-group, mean_30_T-overlap, and ${\text{mean}}_{\text{30}}\bar{\text{F1-score-each}}$) across 30 data segments are listed in S1 Table. We also calculated the TP, FP, FN, recall, precision and VS-F1-score-each values of nEGS-1-each versus EGS-each (see methods in S1 File), and the mean performance (mean_30_TP, mean_30_FP, mean_30_FN, mean_30_Recall, mean_30_Precision and mean_30_VS-F1-score-each) across 30 data segments are shown in S2 Table.

Here, we illustrated that 1) the $\bar{\text{F1-score-each}}$ was the mean F1 score of the group standards (EGS-each and nEGS-1-each) of each data segment at the optimal thresholds; 2) the mean_30_T-group, mean_30_T-overlap and ${\text{mean}}_{\text{30}}\bar{\text{F1-score-each}}$ were the mean T-group, T-overlap and $\bar{\text{F1-score-each}}$ of EGS-each or nEGS-1-each, respectively, across 30 data segments; 3) the VS-F1-score-each was the F1 score of the nEGS-1-each versus the EGS-each; 4) the mean_30_TP, mean_30_FP, mean_30_FN, mean_30_Recall, mean_30_Precision and mean_30_VS-F1-score-each were the mean TP, FP, FN, recall, precision and VS-F1-score-each of the nEGS-1-each versus the EGS-each across 30 data segments, respectively.

Finally, three Pearson’s correlation coefficients were calculated: 1) between the $\bar{\text{F1-score-each}}$ values of EGS-each and nEGS-1-each; 2) between the $\bar{\text{F1-score-each}}$ and VS-F1-score-each of nEGS-1-each; and 3) between the $\bar{\text{F1-score-each}}$ of EGS-each and the VS-F1-score-each of nEGS-1-each. We also performed partial correlation analyses on these variables to assess the specific contribution of each index.

#### The minimum number of non-experts identification spindles

The validity of the method of spindle detection by non-experts was, to some extent, dependent on the number of non-experts assigned to each data segment. A small number of non-experts would be more likely to generate a poor performance. A large number of non-experts would require more time and money to collect their data. A specific procedure described as follows was established to determine the minimum number of non-experts, which considered both accuracy and efficiency (Fig 1D). For each data segment, *n* (*n* = 1,2,3,…,20) non-experts identified spindles. The *n* non-experts were randomly selected from N (range: 21–25) non-experts who identified the data segment. We generated nEGS-1 from the *n* non-experts, obtained the $\bar{\text{F1 score}}(\text{n})$ (the mean F1 score of the nEGS-1 generated from *n* non-experts at the optimal thresholds), and calculated the VS-F1-score(n) by compared nEGS-1 with EGS. The VS-F1-score(n) was the F1 score of nEGS-1, which was generated from the results of *n* non-experts, compared with the EGS. This approach was repeated 500 times. For the *i*th (*i =* 1,2,3,…,500) time, we obtained the $\bar{\text{F1 score}}(\text{n},\text{i})$ and VS-F1-score(n,i) of the nEGS-1 which was generated from the *n* non-experts. The $\bar{\text{F1 score}}(\text{n},\text{i})$ was the mean F1 score of the nEGS-1 generated from *n* non-experts at the optimal thresholds when the approach was repeated for the *i*th time. At the same time, the VS-F1-score(n,i) was the F1 score of the nEGS-1, which generated from the results of *n* non-experts, compared with the EGS.

Then, we calculated the following variables:

$${\text{mean}}_{\text{500}}<\text{VS}-\text{F}1-\text{score}(\text{n})>\;=\;\frac{1}{500}\;\times \;\sum_{\text{i}=1}^{\text{500}}\text{VS}-\text{F}1-\text{score}(\text{n},\text{i}),\text{ n }=\;1,2,3,\ldots ,20$$ (7)

$${\text{mean}}_{\text{500}}<\bar{\text{F1 score}}(\text{n})>\;=\;\frac{\text{1}}{\text{500}}\;\times \;\sum_{\text{i }=1}^{\text{500}}\bar{\text{F1 score}}(\text{n},\text{i}),\text{ n }=\;1,2,3,\ldots ,20$$ (8)

We considered the average of the last three values of mean_500_<VS-F1-score(n)> as the stable value.

$$\text{stable value }=\;\frac{1}{3}\;\times \;\sum_{\text{n }=18}^{\text{20}}{\text{mean}}_{\text{500}}<\text{VS}-\text{F}1-\text{score}(\text{n})>$$ (9)

Finally, we calculated the difference between the mean_500_<VS-F1-score(n)> and the stable value (stable value—mean_500_<VS-F1-score(n)>). The minimum number *n* was determined when the difference was below the threshold of 0.05 for the first time. The selected *n* non-experts were not identical at each repetition; therefore, if ${\text{C}}_{\text{N}}^{\text{n}}$ was less than 500, we only performed ${\text{C}}_{\text{N}}^{\text{n}}$ repetitions. For *n* = 1 and 20, we only performed 21 repetitions with *N* = 21. Similarly, for *n* = 2 and 19, we only performed 210 repetitions.

#### Spindle characterization

After the group standards and automatic standards were established, several spindle characteristics including amplitude, frequency, duration and density were described. After the sleep data was band-pass filtered between 11 and 16 Hz, the frequency (Hz) was calculated by dividing the sampling frequency by the peak-to-peak mean distance within the spindle. The spindle amplitude (μV) was the maximum difference between adjacent local maxima and minima within one spindle. The spindle density was the number of spindles per minute (the total number of spindles divided the length of sleep data).

## Results

### Performance of four group standards

**Fig 2:**
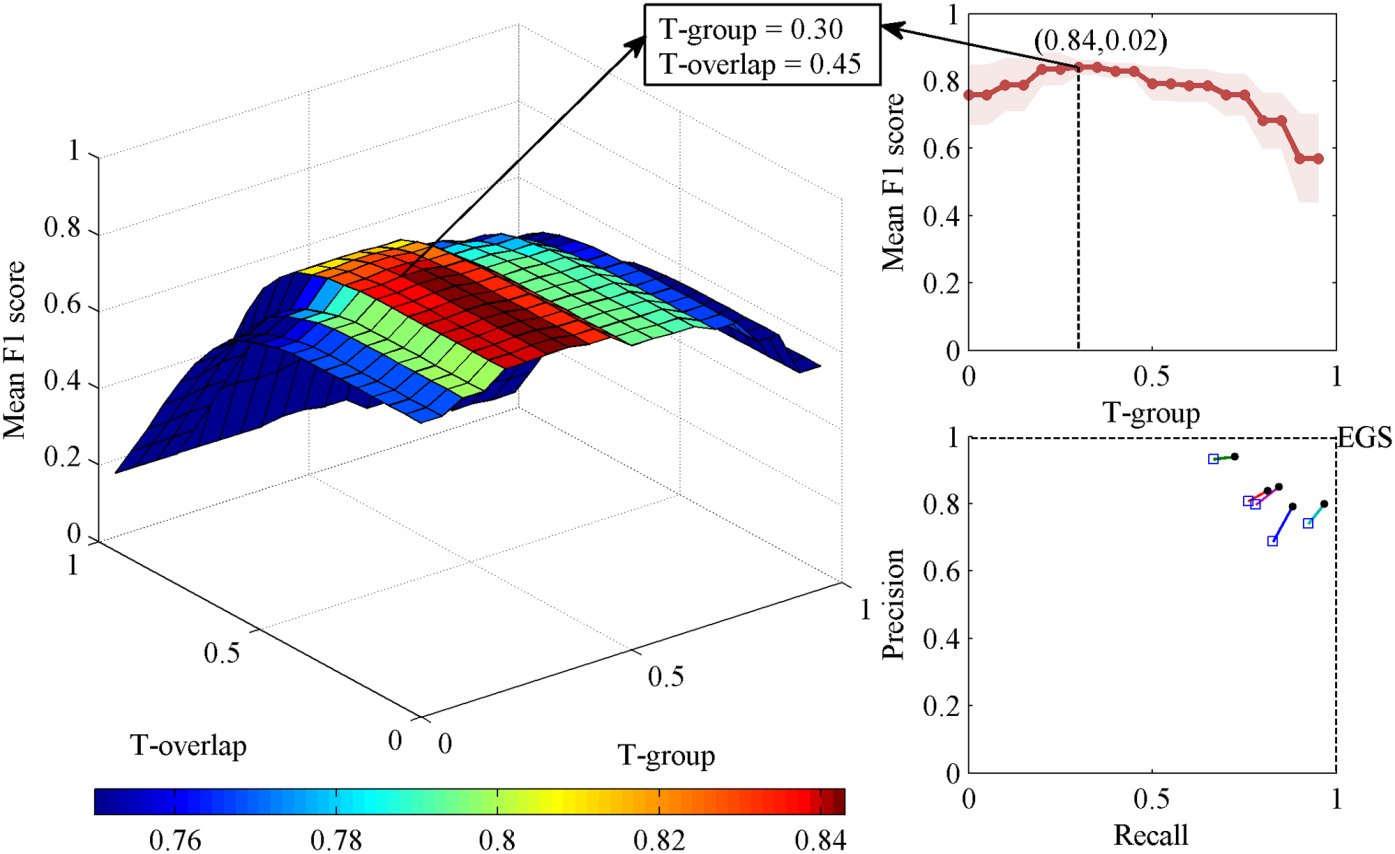
Generation of the expert group standard in stage N2. Left: Three-dimensional plot of the mean F1 score of individual experts at varying group thresholds (T-group) and overlap thresholds (T-overlap). Top right corner: The mean F1 scores of experts at T-overlap = 0.45 with T-groups ranging from 0 to 0.95 at increments of 0.05. The highest average performance was at T-group = 0.30. The shading indicates the standard deviation. At T-group = 0.30 and T-overlap = 0.45, the mean F1 score shows a significant decreasing trend in the direction of the T-overlap axis, and the color clearly changes from dark red to orange (see left). This level of the expert group consensus was the expert group standard (EGS). Bottom right corner: A precision-recall plot of individual expert performance. Each square represents one expert. The square and the dot represent the performance with and without the leave-one-out method.

For stage N2, the EGS was established from expert scorers at the best T-group = 0.30 (Fig 2; Table 1). The F1 score of individual experts ranged from 0.82 to 0.88, and the $\bar{\text{F1 score}}$ was 0.84 ± 0.02 (mean ± standard deviation, throughout) at the optimal thresholds (T-group = 0.30, T-overlap = 0.45). These results indicate good agreement between individual experts and EGS, as well as low variability between experts. The mean performance in the leave-one-out analysis (termed $\bar{\text{F1 score-leave}}$, the mean F1 score of five experts or 168 non-experts in the leave-one-out analysis, see methods in S1 File) was 0.79 ± 0.03. Compared with the $\bar{\text{F1 score}}$, the $\bar{\text{F1 score-leave}}$ showed a slight decrease, which was consistent with the results of Warby et al. [36].

**Fig 3:**
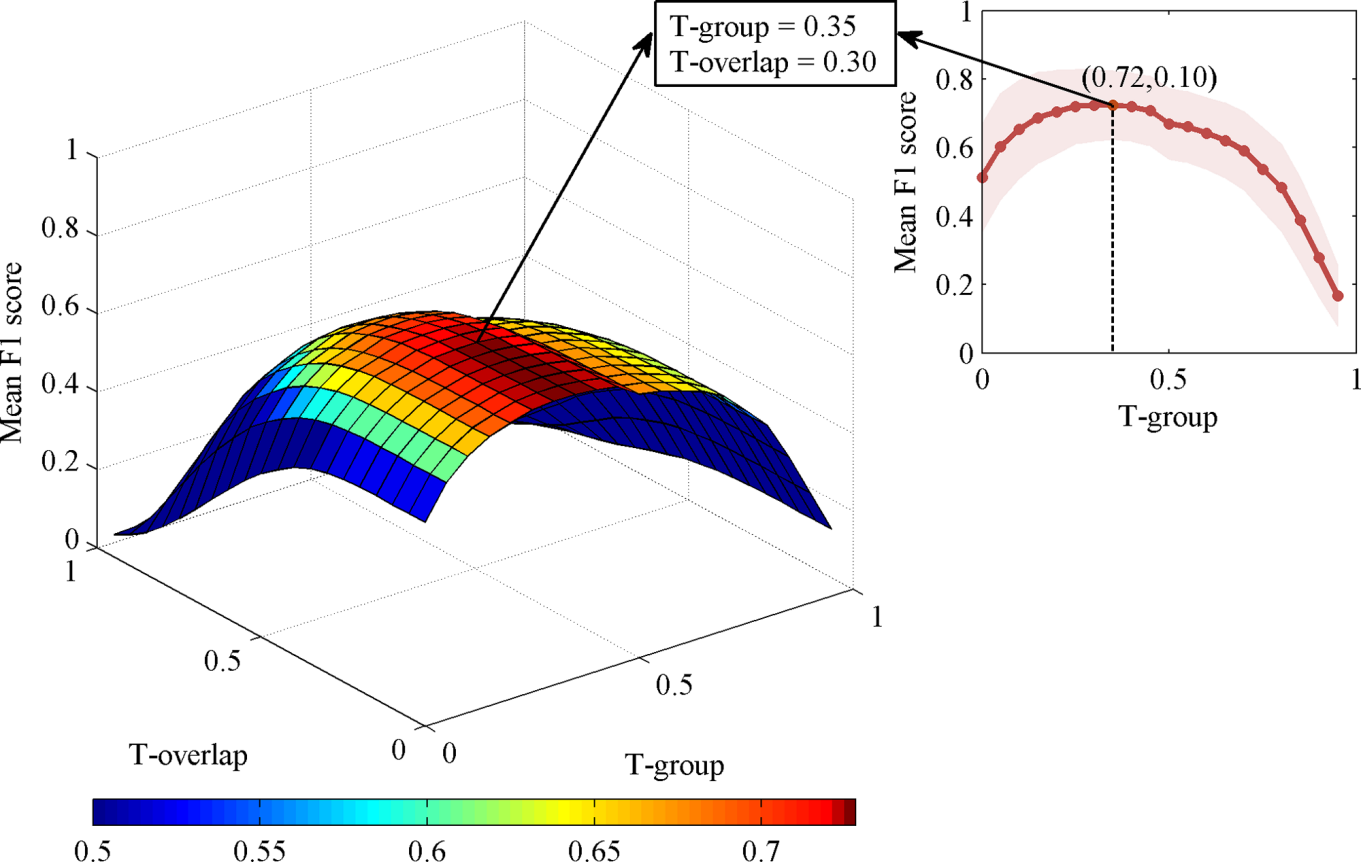
Generation of the non-expert group standard with all spindles in stage N2. Left: Three-dimensional plot of the mean F1 score of individual non-experts at varying group thresholds (T-group) and overlap thresholds (T-overlap) when both definite spindles and indefinite spindles are considered. Top right corner: The mean F1 scores of non-experts at T-overlap = 0.30 with T-groups ranging from 0 to 0.95 at increments of 0.05. The highest average performance was at T-group = 0.35. The shading indicates the standard deviation. At T-group = 0.35 and T-overlap = 0.30, the mean F1 score shows a significant decreasing trend in the direction of the T-overlap axis. This level of the non-expert group consensus was the non-expert group standard with all spindles (nEGS-all).

As shown in Fig 3 and Table 1, the nEGS-all from 168 non-expert scorers was obtained at the best T-group = 0.35 in stage N2 sleep data. The F1 score of individual non-experts ranged from 0.24 to 0.88, and the $\bar{\text{F1 score}}$ was 0.72 ± 0.10 at the optimal thresholds (T-group = 0.35, T-overlap = 0.30), which indicate good agreement between individual non-experts and nEGS-all, as well as low variability between non-experts. Compared with the $\bar{\text{F1 score}}$, the $\bar{\text{F1 score-leave}}$ (0.70 ± 0.10) showed a slight decrease.

In addition, the nEGS-1 and nEGS-05 of stage N2 (Table 1; S1 and S2 Figs) were established. These four group standards of stage N3 were also obtained (Table 1; S3–S6 Figs). Similar results were found in stages N2 and N3: 1) individual experts’ performance was high; 2) individual experts’ results were consistent with EGS; and 3) $\bar{\text{F1 score-leave}}$ was slightly lower than $\bar{\text{F1 score}}$. For both stage N2 and N3 sleep data, the $\bar{\text{F1 score}}$ of nEGS-all was the highest among those of the three non-expert group standards.

To compare the performance of the three non-expert group standards (nEGS-all, nEGS-1 and nEGS-05), we calculated the TP, FP, FN, recall, precision and VS-F1-score by compared them with EGS (Table 2). The results of stage N2 showed that the TP, recall, precision and VS-F1-score of nEGS-1 were close to those of nEGS-all and much higher than those of nEGS-05. Moreover, 138 of 151 TP spindles identified by nEGS-05 were included in the 529 TP spindles identified by nEGS-1. For stage N3, the precision and VS-F1-score of nEGS-1 were slightly higher than those of nEGS-all and much higher than those of nEGS-05. We also found that 23 of the 28 TP spindles identified by nEGS-05 were included in the 135 TP spindles identified by nEGS-1. These findings suggest that nEGS-05 contributes few TP spindles but more FP spindles and that the VS-F1-score of nEGS-1 was slightly higher than that of nEGS-all.

We also measured the performance of the four automated spindle detection methods. For stage N2, the VS-F1-score of AS1 versus EGS was 0.62 at T-overlap = 0.30. However, for stage N3, the VS-F1-score of AS1 was only 0.27 at T-overlap = 0.40, with higher recall but lower precision (Table 2). At T-overlap = 0.35, the VS-F1-scores of AS2 were 0.64 and 0.35 in stages N2 and N3, respectively, the latter of which showed more FP spindles. At T-overlap = 0.30, the VS-F1-scores of AS3 versus EGS were 0.44 and 0.38 in stages N2 and N3, respectively, the latter of which had fewer TP spindles. At T-overlap = 0.25, the VS-F1-scores of AS4 were 0.25 and only 0.08 in stages N2 and N3, respectively, with a large number of FP spindles present. As shown in Table 2, the VS-F1-score of nEGS-1 was higher than those of all automatic methods tested in both stages N2 and N3. The lower VS-F1-scores of the automatic standards in stage N3 sleep data indicate that these automated methods are not suitable for stage N3 sleep data. In summary, compared with EGS, the performance of nEGS-1 was the highest among the three non-expert group standards and also higher than that of the four automatic methods we tested. These results suggest that the nEGS-1, which only uses the weighted value of 1 for the analysis, is suitable to be the final non-expert group standard.

**Fig 4:**
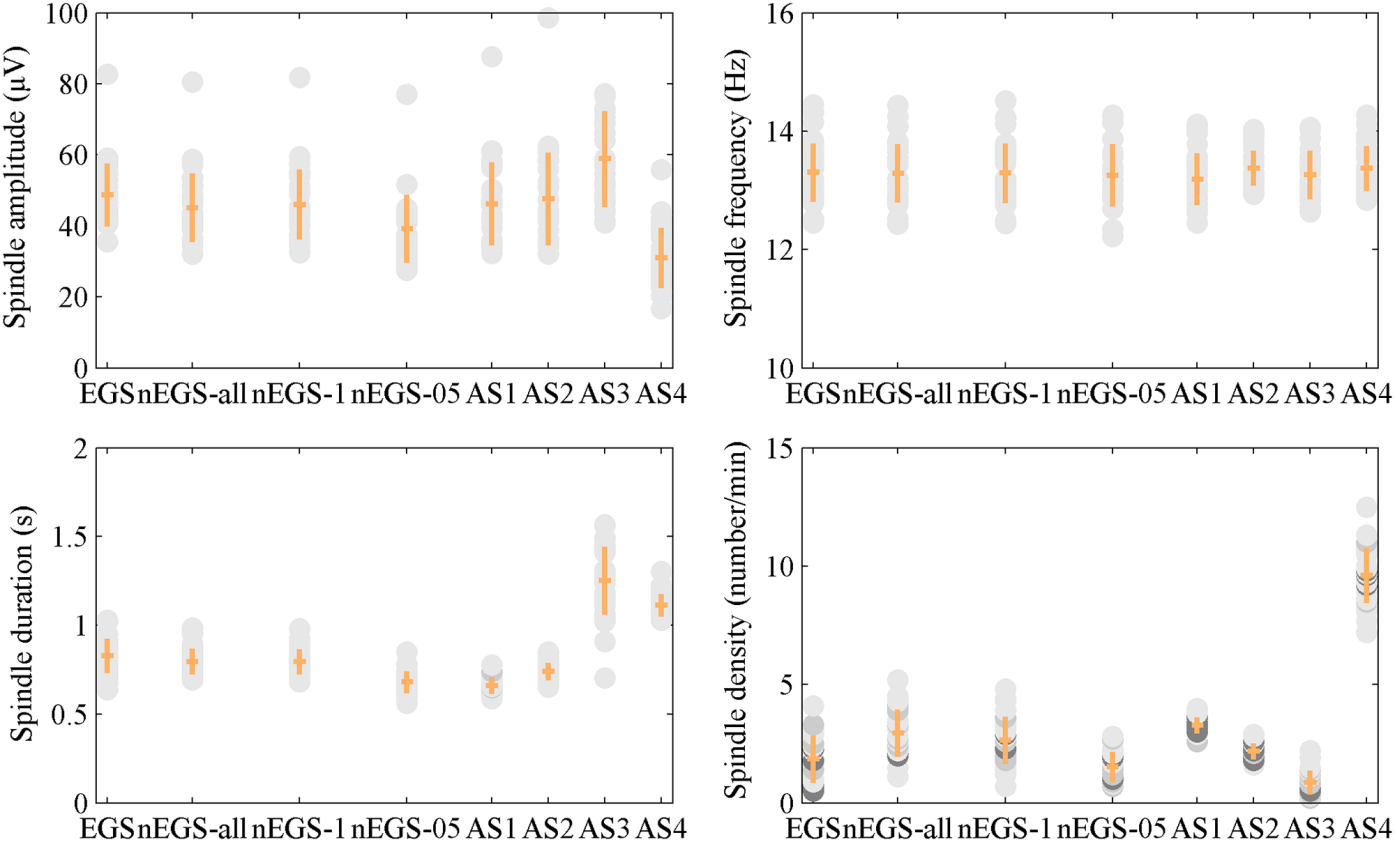
Spindle characteristics for each standard of stage N2. (A) Spindle amplitude, (B) frequency, (C) duration and (D) density. Each dot is one subject (n = 30). The mean and standard deviation of each standard are indicated by orange horizontal and vertical lines. The EGS was the expert group standard. The nEGS-all was the non-expert group standard with all spindles. The nEGS-1 was the non-expert group standard with definite spindles. The nEGS-05 was the non-expert group standard with indefinite spindles. The AS1, AS2, AS3 and AS4 were the automatic standards obtained by the first, second, third and fourth automated method, respectively.

**Fig 5:**
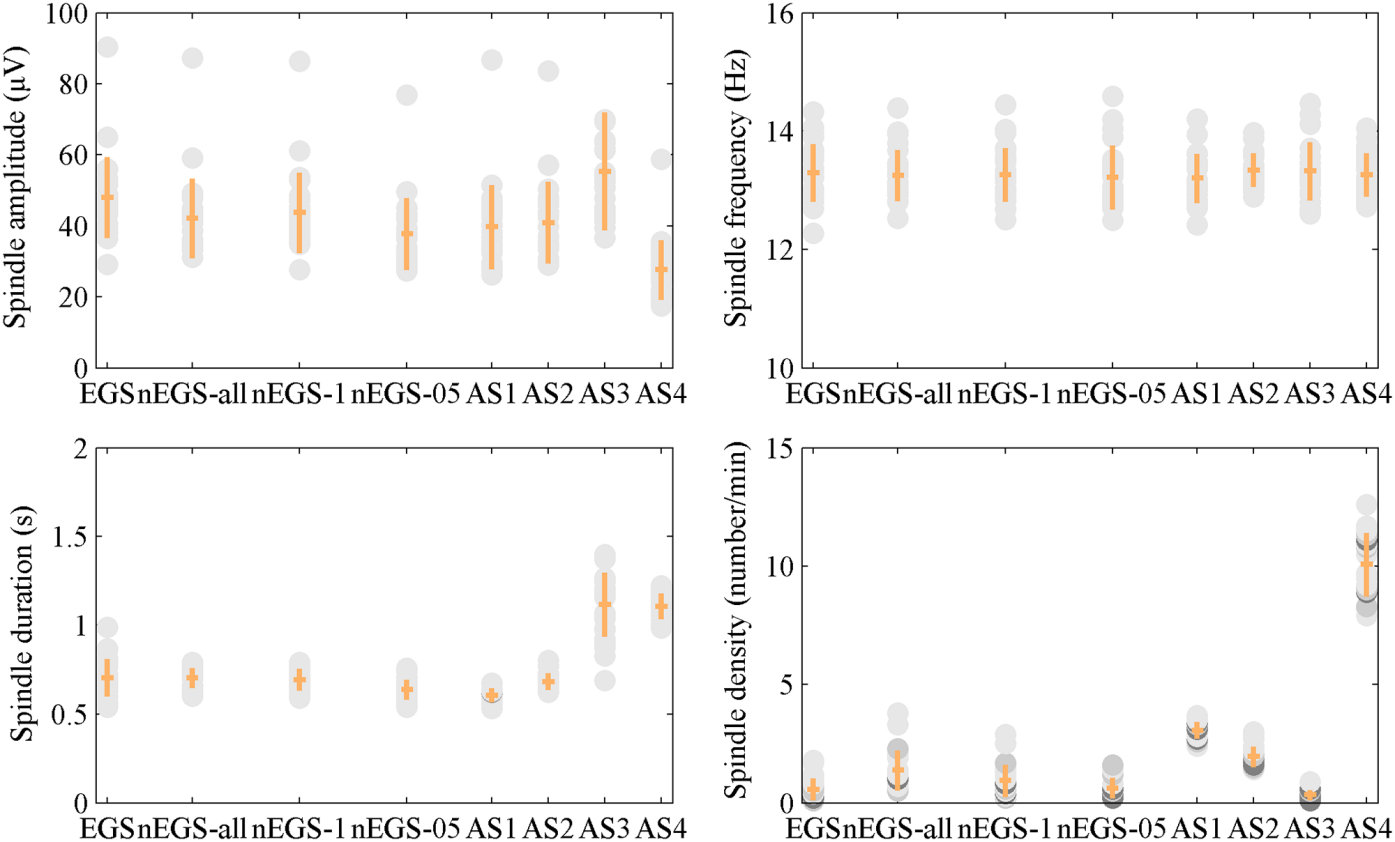
Spindle characteristics for eight standards of stage N3. (A) Spindle amplitude, (B) frequency, (C) duration and (D) density. The eight standards are EGS (expert group standard), nEGS-all (non-expert group standard with all spindles), nEGS-1 (non-expert group standard with definite spindles)**,** nEGS-05 (non-expert group standard with indefinite spindles), AS1, AS2, AS3 and AS4 (automatic standards obtained by the first, second, third and fourth automated method, respectively). Each dot is one subject (n = 30). The mean and standard deviation of each standard are indicated by orange horizontal and vertical lines.

On the other hand, we explored the spindle characteristics: spindle amplitude (μV), spindle frequency (Hz), spindle duration (s) and spindle density. For stage N2 sleep data, the spindles identified by nEGS-all and nEGS-1 differed from those identified by EGS (Table 3) in terms of spindle amplitude (P < 10^−5^), duration (P < 0.05) and density (P < 10^−8^), but not mean frequency (P > 0.10). For stage N3 sleep data, they differed from those identified by EGS in terms of spindle amplitude (P < 10^−4^) and density (P < 10^−5^), but not mean frequency (P > 0.05) or duration (P > 0.10). For both stages N2 and N3, spindles identified by nEGS-05 differed from those identified by EGS in terms of spindle amplitude (P < 10^−8^), and duration (P < 0.05), but not mean frequency (P > 0.05) or density (P > 0.10). Spindles identified by AS1 were smaller than those identified by EGS in terms of spindle amplitude (P < 0.05), frequency (P < 0.05) and duration (P < 10^−3^), but were larger in terms of spindle density (P < 10^−8^), as for both stage N2 and N3 sleep data. The duration of spindles identified by AS2 was less than that of those identified by EGS in stage N2 sleep data (P < 10^−6^), but there was no difference in stage N3 sleep data (P > 0.10). Spindles identified by AS3 and AS4 differed from those identified by EGS in terms of amplitude (P < 10^−4^), duration (P < 10^−10^) and density (P < 0.05), but not mean frequency (P > 0.10), as for both stage N2 and N3 sleep data. Despite these differences, the spindle characteristics of EGS and nEGS-1 were similar (Figs 4 and 5), further verifying the conclusion that the nEGS-1, which is independent of expert, is suited as the final non-expert group standard.

**Table 3:** Spindle characteristics for automatically and manually detected spindles by groups of experts and non-experts.

Stage	Standard	Amplitude(μV)	Frequency(Hz)	Duration(s)	Density
**N2**	**EGS**	48.72 ± 8.81	13.31 ± 0.49	0.83 ± 0.10	1.85 ± 0.10
	**nEGS-all**	45.14 ± 9.59^a^	13.29 ± 0.49	0.80 ± 0.07^a^	2.94 ± 0.99^a^
	**nEGS-1**	46.06 ± 9.75^a^^b^	13.29 ± 0.50	0.79 ± 0.07^a^	2.65 ± 0.99^a^^b^
	**nEGS-05**	39.27 ± 9.52^a^^b^^c^	13.26 ± 0.52	0.68 ± 0.06^a^^b^^c^	1.51 ± 0.66^b^^c^
	**AS1**	46.21 ± 11.60^a^^d^	13.19 ± 0.44^a^^b^^c^	0.66 ± 0.05^a^^b^^c^	3.27 ± 0.33^a^^c^^d^
	**AS2**	47.68 ± 13.00^b^^c^^d^^e^	13.38 ± 0.29^b^^d^^e^	0.74 ± 0.05^a^^b^^c^^d^^e^	2.18 ± 0.33^b^^c^^d^^e^
	**AS3**	58.89 ± 13.42^a^^b^^c^^d^^e^^f^	13.26 ± 0.41^f^	1.25 ± 0.19^a^^b^^c^^d^^e^^f^	0.86 ± 0.49^a^^b^^c^^d^^e^^f^
	**AS4**	31.03 ± 8.37^a^^b^^c^^d^^e^^f^^g^	13.37 ± 0.37^b^^c^^d^^e^^g^	1.11 ± 0.06^a^^b^^c^^d^^e^^f^^g^	9.61 ± 1.15^a^^b^^c^^d^^e^^f^^g^
**N3**	**EGS**	47.78 ± 11.04	13.37 ± 0.51	0.69 ± 0.11	0.51 ± 0.45
	**nEGS-all**	41.48 ± 11.08^a^	13.24 ± 0.47	0.69 ± 0.06	1.23 ± 0.87^a^
	**nEGS-1**	43.59 ± 11.00^a^^b^	13.25 ± 0.46	0.69 ± 0.06	0.83 ± 0.69^a^^b^
	**nEGS-05**	37.29 ± 9.64^a^^b^^c^	13.20 ± 0.54	0.64 ± 0.06^a^^b^^c^	0.57 ± 0.41^b^^c^
	**AS1**	38.87 ± 11.36^a^^b^^c^^d^	13.23 ± 0.41^a^	0.60 ± 0.04^a^^b^^c^^d^	3.06 ± 0.34^a^^b^^c^^d^
	**AS2**	40.11 ± 10.94^a^^c^^d^^e^	13.37 ± 0.30^b^^e^	0.68 ± 0.04^d^^e^	1.92 ± 0.42^a^^b^^c^^d^^e^
	**AS3**	54.95 ± 16.02^a^^b^^c^^d^^e^^f^	13.36 ± 0.57^e^	1.10 ± 0.19^a^^b^^c^^d^^e^^f^	0.31 ± 0.21^a^^b^^c^^d^^e^^f^
	**AS4**	27.09 ± 8.11^a^^b^^c^^d^^e^^f^^g^	13.29 ± 0.36	1.11 ± 0.07^a^^b^^c^^d^^e^^f^	9.91 ± 1.37^a^^b^^c^^d^^e^^f^^g^

Data are presented as mean ± standard deviation unless otherwise indicated. The EGS was the expert group standard. The nEGS-all was the non-expert group standard with all spindles when both the weighted value of 1 and 0.5 were considered. The nEGS-1 was the non-expert group standard with definite spindles when the weighted value of 1 was only considered. The nEGS-05 was the non-expert group standard with indefinite spindles when the weighted value of 0.5 was only considered. The AS1, AS2, AS3 and AS4 were the automatic standards obtained by the first, second, third and fourth automated method, respectively.

^a^Indicates significant difference from EGS.

^b^Indicates significant difference from nEGS-all.

^c^Indicates significant difference from nEGS-1.

^d^Indicates significant difference from nEGS-05.

^e^Indicates significant difference from AS1.

^f^Indicates significant difference from AS2.

^g^Indicates significant difference from AS3.

### Performance of group standards of each data segment

For each data segment, we established the EGS-each and nEGS-1-each, and obtained the best T-group, T-overlap and $\bar{\text{F1-score-each}}$. A comparison of the results in Table 1 with those in S1 Table shows that, for stage N2, the ${\text{mean}}_{\text{30}}\bar{\text{F1-score-each}}$ of EGS-each was 0.83. That value was extremely close to the $\bar{\text{F1 score}}$ of EGS. The ${\text{mean}}_{\text{30}}\bar{\text{F1-score-each}}$ of nEGS-1-each was also close to the $\bar{\text{F1 score}}$ of nEGS-1. Similar findings were obtained in stage N3. We also found that for stage N2 sleep data, the VS-F1-score of EGS-each compared with EGS was 0.97, and the VS-F1-score-nEGS-1 (the F1 score of nEGS-1-each versus nEGS-1) was 0.96. Likewise, for stage N3, the VS-F1-score of EGS-each was 0.93, and the VS-F1-score-nEGS-1 of nEGS-1-each was 0.90. These results showed that the performance of group standards generated from each data segment was highly consistent with that generated from all data. These findings indicate that the method of generating group standards is not affected by dataset length.

A comparison of the results in stage N2 in Table 2 with those in S2 Table shows that, the mean_30_VS-F1-score-each of nEGS-1-each was 0.77 ± 0.15 (i.e., close to the VS-F1-score of nEGS-1). For stage N3, similar results were obtained; the mean_30_VS-F1-score-each of nEGS-1-each was 0.63 ± 0.14, and the VS-F1-score of nEGS-1 was 0.68. These results were expected because of the high consistency between nEGS-1-each and nEGS-1, and they further verified the conclusion that dataset length does not influence the validity of the method for generating group standards.

**Fig 6:**
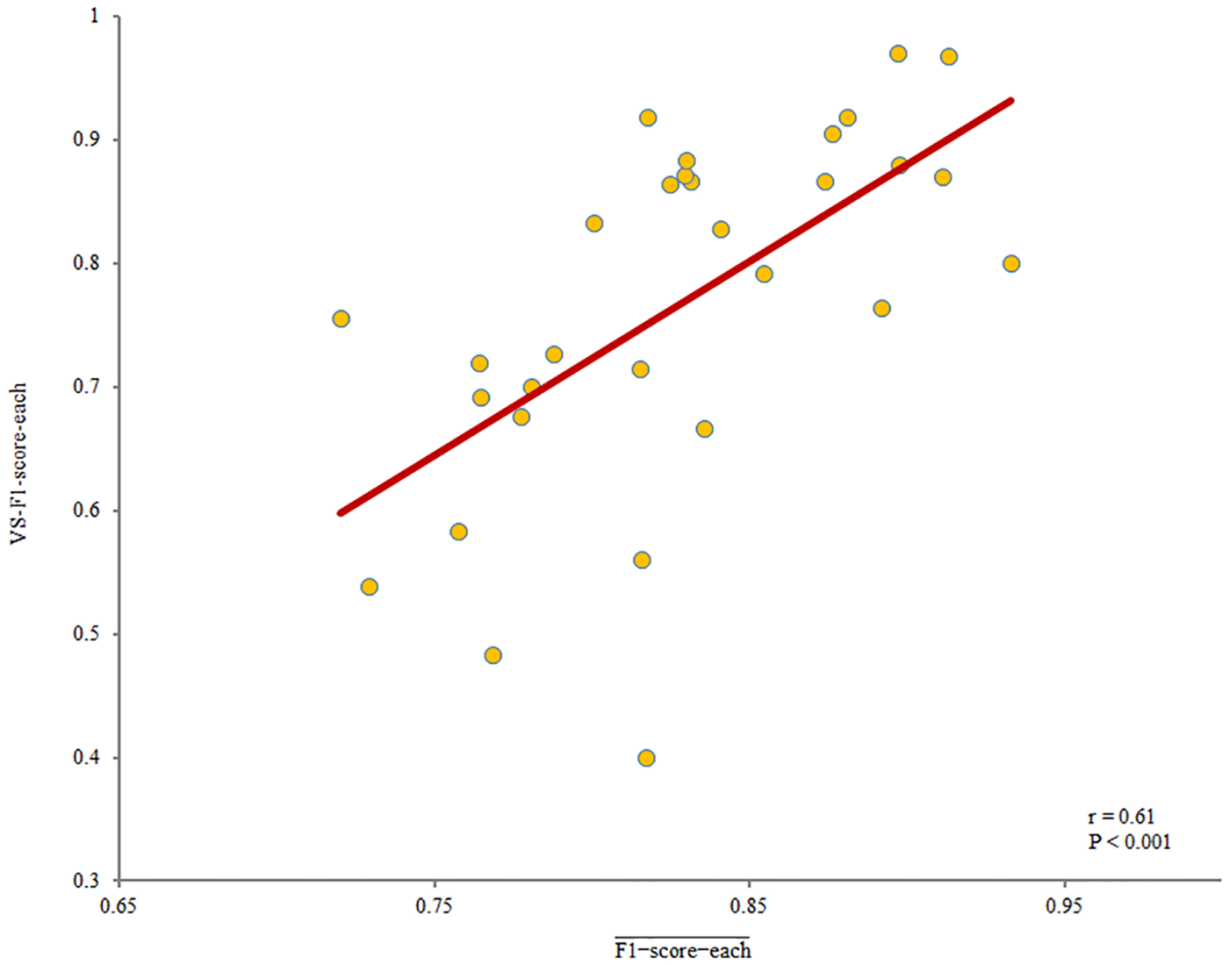
Correlation between $\bar{\text{F1-score-each}}$ of EGS-each and VS-F1-score-each of nEGS-1-each in stage N2 sleep data. Note that after remove one outlier, the $\bar{\text{F1-score-each}}$ of EGS-each was still significantly correlated with the VS-F1-score-each of nEGS-1-each (r = 0.67, P < 10^−4^). The EGS-each is the expert group standard of each data segment. The nEGS-1-each is the non-expert group standard with definite spindles of each data segment. The $\bar{\text{F1-score-each}}$ was the mean F1 score of EGS-each of each data segment at optimal thresholds. The VS-F1-score-each was the F1 score of nEGS-1-each versus EGS-each.

For stage N2, Pearson’s correlation coefficients showed positive relationships between the $\bar{\text{F1-score-each}}$ values of EGS-each and nEGS-1-each (r = 0.56, P < 0.01), between the $\bar{\text{F1-score-each}}$ of EGS-each and the VS-F1-score-each of nEGS-1-each (r = 0.61, P < 0.001; Fig 6), and between the $\bar{\text{F1-score-each}}$ and VS-F1-score-each of nEGS-1-each (r = 0.53, P < 0.01). However, a partial correlation analysis revealed a statistically significant relationship only between the $\bar{\text{F1-score-each}}$ of EGS-each and the VS-F1-score-each of nEGS-1-each (r = 0.44, P < 0.05; Fig 6). For stage N3, Pearson’s correlation analysis and partial correlation analysis found no correlations among these scores. These results suggest that the data segment with higher performance of experts also shows higher performance of non-experts only in stage N2.

#### The minimum number of non-experts identification spindles

**Fig 7:**
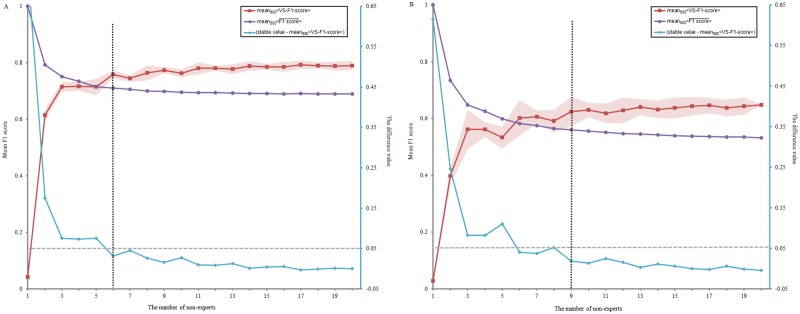
The performance of nEGS-1 with different numbers of non-experts identifying one sleep data. (A) stage N2, (B) stage N3. n is the number of non-experts (n = 1,2,3,…,20). The red square indicates the mean performance of nEGS-1 versus EGS over 500 repetitions (mean_500_<VS-F1-score(n)>). The shading indicates the standard deviation. The purple circle indicates the mean F1 score of non-experts in nEGS-1 at the optimal thresholds over 500 repetitions (${\text{mean}}_{\text{500}}<\bar{\text{F1 score}}(\text{n})>$). The blue diamond (right ordinate) indicates the difference between the mean_500_<VS-F1-score(n)> of nEGS-1 and the stable value of the mean performance of the last three values in the red square line (stable value -mean_500_<VS-F1-score(n)>). The nEGS-1 is the non-expert group standard with definite spindles. The EGS is the expert group standard.

Because the nEGS-1 had been identified as the final non-expert group standard according to the above results, we only considered the performance of nEGS-1 when determining the minimum number of non-experts for reliable spindle identification. Fig 7 illustrates the performance of nEGS-1 with different numbers of non-experts. For stage N2, the mean performance of nEGS-1 versus EGS over 500 repetitions (i.e., mean_500_<VS-F1-score(n)>, n = 1,2,3,…,20) tended to stabilize with an increased number of non-experts. The stable value was 0.79 by averaging the last three values of mean_500_<VS-F1-score(n)>. The difference between the stable value and mean_500_<VS-F1-score(n)> of nEGS-1 was less than 0.05 when the number of non-experts ranged from 6 to 20 (Fig 7A). Thus, for stage N2 sleep data, at least six non-experts seem to be required to identify spindles. For stage N3, the stable value was 0.64, and the difference value was less than 0.05 when the number of non-experts ranged from 9 to 20 (Fig 7B). Thus, we recommend that at least nine non-experts should be employed for spindle identification in each stage N3 sleep data segment.

To check the robustness of these results, we also determined the minimum number of non-experts using the k-means clustering method. We divided 8462 F1 scores into 2 clusters (S5 Table). For stages N2 and N3, the centroids of Cluster 1 were 0.69 and 0.52, respectively, with low performance. The centroids of Cluster 2 were 0.78 and 0.63 in stages N2 and N3, respectively, with high performance. S5 Table shows that for stage N2, when 6–20 non-experts identified spindles, at least 80% of the F1 scores in each condition were classified into Cluster 2, with high performance. For stage N3, more than 80% of F1 scores in each condition were classified into Cluster 2 when 10–20 non-experts identified spindles. These results are consistent with the minimum numbers of non-experts which were found by statistical methods.

We also determined the minimum number of experts using methods similar to those for non-experts. For each data segment, *n* (*n* = 1,2,3,4) experts who were randomly selected from a pool of 5 experts identified spindles. We generated the expert group standard from these *n* experts, and calculated the VS-F1-score(n) by compared with the EGS. This approach was repeated ${\text{C}}_{5}^{\text{n}}$ times. The mean VS-F1-score(n) over ${\text{C}}_{5}^{\text{n}}$ repetitions is presented in S6 Table, which shows a maximal rate of increase with 3 experts identifying sleep spindles in both stages N2 and N3. Moreover, the performance of 3 experts was acceptable (0.92 and 0.84 for N2 and N3, respectively). Thus, we recommend that for both stages N2 and N3 at least 3 experts should be employed to identify spindles.

## Discussion

The purpose of our study was to explore an approach for accurate and efficient detection of sleep spindles by a group of non-experts in a crowdsourcing scheme. Although Warby et al. [36] indicated that spindle identification by non-experts is viable as a spindle detection method, Warby’s process of establishing a non-expert group consensus was based on EGS, which hindered the application of this spindle detection method. To overcome this limitation, first, we described a method of optimizing the thresholds of the non-expert group consensus which was based on the data from non-experts themselves. Using this method, we established three non-expert group standards (nEGS-all, nEGS-1 and nEGS-05) from non-experts and generated the EGS from experts. Compared with the EGS, the performance of nEGS-1 was the highest among the three non-expert group standards, and also higher than the four automatic methods we tested; therefore, we identified the nEGS-1 as the final non-expert group standard. These findings suggest that this approach to generating non-expert group standard, independent of expert, is suitable. It is also suggested that its performance would be higher when definite spindles are only considered. Second, data segments with better performance by experts also showed better performance by non-experts in stage N2. Better scorer performance may reflect the presence of more obvious, easily identifiable attributes (e.g., larger EEG amplitudes or less background activity). These results suggest that spindle identification is affected by intrinsic data properties that are related to the difficulty level of spindle identification, especially for stage N2 sleep data. Third, the performance of nEGS-1 compared with the EGS was acceptable when there were six and nine non-experts identifying stage N2 and N3 sleep data, respectively.

### Non-expert group standards: Independent of EGS

The non-expert group consensus in the study by Warby et al. [36] and Ray et al. [37] was obtained by maximizing the non-expert group’s performance in comparison with EGS. Therefore, for practical application of this method, establishment of a non-expert group standard that is independent of experts is necessary. To our knowledge, few studies that establish the non-expert group standard independent of experts have been reported.

In the present study, we generated the nEGS-all with a modified version of Warby’s method. To generate the non-expert group standard, we determined the optimal T-group and T-overlap. The optimal T-group was determined by maximizing the mean F1 score of all individuals in the non-expert group rather than maximizing the F1 score of the non-expert group consensus versus EGS [36, 37]. Each group standard had its own optimal T-overlap, instead of a constant threshold; thus, the non-expert group standard established by our procedure is generated by using only data from non-experts themselves (i.e., independent of experts).

Furthermore, we distinguished between the confidence values of 1 and 0.5, which was not done in previous studies, and generated nEGS-1 and nEGS-05 using the same modified method. The results showed that for both N2 and N3 sleep data the mean F1 score and the F1 score range of nEGS-1 were close to those of nEGS-all (Table 1). However, the $\bar{\text{F1 score}}$ of nEGS-05 was much lower than that of both nEGS-all and nEGS-1, and the F1 score range showed large differences between nEGS-05 and the other two non-expert group standards. These findings suggest that the consistency of the performance of individual non-experts in nEGS-1 was almost as high as that in nEGS-all, and much higher than that in nEGS-05.

The three non-expert group standards were also compared with the EGS. We found that for both N2 and N3 sleep data the VS-F1-score of nEGS-1 was slightly higher than that of nEGS-all, and much higher than that of nEGS-05 (Table 2). These results suggest that nEGS-1 was the most consistent with EGS. For stage N2, 138 of 151 TP spindles identified by nEGS-05 were among the 529 TP spindles identified by nEGS-1, but only 84 of 303 FP spindles identified by nEGS-05 were among the 265 FP spindles identified by nEGS-1. Similar results were obtained for stage N3: a majority of TP spindles identified by nEGS-05 were also identified by nEGS-1, but only a small proportion of the FP spindles identified by nEGS-05 were also identified by nEGS-1. These findings indicate that nEGS-05 contributes few TP spindles but more FP spindles in both stage N2 and N3.

In summary, 1) individual non-experts’ mean performance in nEGS-1 was almost as good as that in nEGS-all, and was much higher than that in nEGS-05; 2) compared with the EGS, the performance of nEGS-1 was the highest among the three non-expert group standards; 3) the nEGS-05 contributed few TP spindles but more FP spindles. These findings suggest that the weight of 1 should be only considered to obtain the non-expert group standards.

The spindle density in nEGS-1 of stage N2 sleep data ranged from 0.70 to 4.80 n/min, and the mean spindle density was 2.65 ± 0.99 n/min, which are in the normal range [43, 44]. All spindles in nEGS-1 were between 0.5 and 1 s in duration, and the mean duration was 0.79 ± 0.07 s conforming to AASM guidelines [1]. The mean frequency of spindles was 13.29 ± 0.50 Hz, and the mean amplitude of spindles was 46.06 ± 9.75μV, which conform to the extant literature [45, 46]. These results suggest that nEGS-1 has good external validity with respect to the extant literature in terms of spindle characteristics.

Experts’ performance has been previously discussed. Wendt et al. [47] found that four or more experts were needed to generate an ‘almost perfectly’ reliable sleep spindle dataset. In our study, we recruited five experts. Additionally, for stage N2, the $\bar{\text{F1 score}}$ of the five experts was consistently high and showed less variability (Table 1). S3 Table shows that the pair-F1-score (the F1 score of inter-expert) ranged from 0.69 to 0.78 among the experts (see methods in S1 File), and the $\bar{\text{pair-F1-score}}$ (the mean pair-F1-score among the experts) was 0.73 ± 0.03. Three studies using 2–3 experts and healthy subjects (aged 21–59 years) found average inter-expert agreements of 86% [48], 70 ± 8% [23] and 81% [30]. However, it is unclear what measurements of agreement are reported. One study using patients with various sleep pathologies of age 31–54 years found considerably lower inter-expert agreement of 46% in sleep spindle scoring [31]. Moreover, Wendt et al. [47] found that the average inter-expert F1 score agreement was 61 ± 6% (ranging from 46% to 74%). Our estimate of 0.73 ± 0.03 inter-expert agreement falls in the middle of this range and our range of the pair-F1-score of 0.69–0.78 fits well with previously reported numbers of agreement between one expert pair. For stage N3, the pair-F1-score ranged from 0.48 to 0.68, and the $\bar{\text{pair-F1-score}}$ was 0.58 ± 0.07. These findings suggest that our expert group standard may produce reliable sleep spindle detection results, especially for stage N2 sleep data. A non-expert group consensus (also called non-expert group standard dependent of expert) was established in stage N2 and N3 sleep data similar to how one was established by Warby et al. [36] (S1 File). For stage N2, the VS-F1-score of the non-expert group standard dependent of expert versus EGS was higher than those of the four automatic methods and slightly lower than the $\bar{\text{F1 score}}$ of EGS; these findings are consistent with those of Warby et al. [36].

#### The mean performance of EGS and nEGS-1

The $\bar{\text{F1 score}}$ of EGS and nEGS-1, as well as the VS-F1-scores of nEGS-1 and four automatic methods, were calculated at varying overlap thresholds (S7 Fig). For both stage N2 and N3, the $\bar{\text{F1 score}}$ of EGS was higher than that of nEGS-1, indicating that experts are more likely than non-experts to reach a consensus on spindle scoring. We also found that the $\bar{\text{F1 score}}$ of EGS and nEGS-1 were higher in stage N2 than N3. One possible reason is that stage N3 sleep spindles are usually superimposed on slow waves, which increases the difficulty of human spindle identification. Furthermore, the stage N2 $\bar{\text{F1 score}}$ of EGS in the present study was higher than that in Warby’s study (0.84 ± 0.02 vs 0.75 ± 0.06 in our study and Warby et al., respectively). This discrepancy may be caused by the age difference of the subjects who provided the EEG data set. The present study’s EEG data set was acquired from young subjects, who have relatively large spindle amplitude and stable shape. However, in the study by Warby et al., the spindle amplitude and duration of the middle-and older-aged subjects were on the decline [39, 49], which may have impaired spindle identification performance. Further, the number of recruited experts in our study was much lower than that by Warby et al. (5 vs 24 in our study and Warby et al., respectively). The possibility of achieving high consistency among experts was increased with the reduction in their number. These findings suggest that the performance of experts and non-experts in the present work is affected by intrinsic features of the EEG data. More discussion of this effect is presented below.

#### The performance of nEGS-1 and automated methods versus EGS

S7 Fig shows that the VS-F1-score of nEGS-1 was higher than those of the four automatic methods in both stage N2 and N3 sleep data. These results indicate that a large group of non-experts may be more useful than several automated spindle algorithms for spindle detection. The VS-F1-scores of the four automatic methods were much lower in stage N3 than N2 sleep data. Additionally, the difference between the VS-F1-score of nEGS-1 and those of the four automatic standards (the VS-F1-score of nEGS-1 minus that of the four automatic standards, except the AS3) was greater in stage N3 than N2. These results suggest that non-experts were superior to these automated spindle algorithms at detecting sleep spindles in stage N3 sleep data. For stage N2, we also found that the VS-F1-scores of the four automatic methods in our study were higher than those in Warby et al. (except the fourth automatic method). This discrepancy may also have resulted from the EEG data set. We collected the EEG data from younger subjects while Warby from older subjects. This finding suggests that age-related decline in spindle amplitude may impair the performance of these amplitude threshold-based algorithms.

#### Performance of scorers manual spindle scoring: Data dependency

Our results indicate that the performance of spindle identification in stage N2 was superior to that in stage N3, with the latter having a lower mean F1 score and larger variance (Tables 1 and 2). One possible explanation for this observation is that the sleep data of stage N3 are characterized by slow waves, which increases the difficulty of human spindle identification. We also found a wide range among the F1 scores of 168 non-experts in nEGS-1 (Table 1), which may have resulted not only from the skill level of the non-experts but also from the inherent nature of the data. The differences caused by the skill level of the non-experts could be overcome by averaging, whereas the differences arising from intrinsic EEG data features (e.g., thicker skull thickness resulting in lower EEG amplitude) may be more difficult to solve.

Thus, we examined the stability of nEGS-1’s performance across different data segments by compared with that of EGS. To address this question, we established EGS-each and nEGS-1-each. Our results showed that for stage N2, data with greater consistency between individual experts in EGS-each had a higher performance of nEGS-1-each (Fig 6). These findings suggest that for stage N2 sleep, easily identifiable visual data would yield better performance by both experts and non-experts.

However, our results from stage N3 sleep data did not support this correlation between experts and non-experts. This discrepancy may be caused by the number of sleep spindles identified in stage N3 sleep data which was significantly lower than that in stage N2 sleep data [50], and by the greater difficulty of sleep spindle identification in stage N3 sleep data which is characterized by slow waves. For these reasons, the present study is applicable mainly for the N2 stage and we should cautious when applying this spindle detection method to stage N3 sleep data. Further research is expected to verify our findings in stage N3. A high-pass filter would reject slow waves and improve correct spindle detection in stage N3.

#### The minimum number of non-experts identification spindles

Samples in sleep research generally comprise approximately 20–30 subjects per group. The mean duration of stage N2 for each subject was approximately 3 hours for the entire night, and the mean duration of stage N3 was 90 min [51]. In total, there were 90 and 45 hours of stage N2 and N3 data, respectively, for 30 subjects. In our study, after studying and practicing, one non-expert took an average of approximately 20 min to identify sleep spindles in per 10-min data segment. Each data segment was identified by at least 20 non-experts. Each day, 15 non-experts identified sleep spindles in our special laboratory. If each non-expert finished six segments of data (60 min of data in total) daily, we would need 540 non-experts to spend 120 days to collect sleep spindle data from 90 hours of stage N2 data, and we would need 270 non-experts to spend 60 days identifying 45 hours of stage N3 data from 30 subjects. We determined that at least six and nine non-experts are required to identify one stage N2 and N3 sleep data segment, respectively (Fig 7). Thus, we would only need 162 non-experts to spend 36 days to identify spindles in 90 hours of stage N2 data and 122 non-experts to spend 27 days to finish 45 hours of stage N3 data, greatly reducing the time and economic costs. For the computational resources, the time complexity was O(m*n), where *m* was the number of non-experts identifying each data segment, and *n* was the number of data segments. These results suggest that this method is efficient and worthy of being generalized for both stage N2 and stage N3 sleep data.

Although the number of non-experts needed to identify spindles and the amount of time needed to collect the non-expert data were acceptable, we would need more non-experts to spend more time with the overall rapid growth in biological and clinical research on sleep spindles. An internet interface for spindle identification that is not restricted to non-experts in laboratories can be developed. Such an internet interface could remotely collect non-expert data so that countless non-experts could perform their tasks simultaneously. Therefore, an internet interface might collect sleep spindle data sets from non-experts more rapidly.

### Limitations

Several limitations of this work should be discussed. First, although we extracted the EEG data set from 30 subjects, only 10 min of data were selected from each subject. The small quantity of data from each subject may have caused some of their characteristics (e.g., spindle density) to be represented less accurately. Second, topographical differences between sleep spindles have been shown [52]. Spindle characteristics and morphology differ between channels, leading to variations in human performance. We only analyzed the C3-M2 channel; the performance of this method in different channels is unclear. Third, the recall of nEGS-1 was relatively high in both stages N2 and N3. However, in both cases, the precision was low. These findings indicate that the non-experts seem to identify spindles easily and erroneously, with a large number of false positive spindles. Indeed, a perfect scorer would fall in the top right corner of a precision-recall plot. Some scorers emphasize high precision at the expense of recall, with few true positives and more false negatives (for example, AS3) and vice versa (for example, AS4, see Table 2). To balance these two measures, we applied the F1 score, which is the harmonic mean of precision and recall. Table 2 shows that the VS-F1-score of nEGS-1 is better than those of automatic methods, with more true positives and fewer false negatives. To summarize, although nEGS-1 present many false positives, their precision was acceptable under these conditions. Fourth, our work was performed among healthy young subjects. It seems to be difficult to prove that our method is useful in clinical data sets or in older subjects. Actually, before the non-experts would start identifying spindles in clinical data sets and in older subjects, they would have to learn how to accurately identify spindles in these unhealthy and older subjects, and have performed a training session. In other words, for different types of data sets, non-experts would be instructed to read the corresponding manual on spindle identification. Thus, we think that our method would be applied to clinical data sets and older subjects. Further studies are expected to test these hypotheses.

### Conclusions

This study established an approach to facilitate accurate and efficient detection of sleep spindles by non-experts. First, a certain number of non-experts should be recruited to identify sleep spindles. We recommend that at least six or nine non-experts score stage N2 and N3 sleep data, respectively. Each non-expert studied a manual on spindle identification and was trained by completing some exercises. Second, although the non-experts were required to distinguish between definite sleep spindles and indefinite sleep spindles, only definite sleep spindles were used to obtain the non-expert group standard. The performance of this method was slightly lower than that of the experts but higher than that of several commonly used automated methods. We also found that easily identifiable data showed higher performance by non-experts. Therefore, crowdsourcing with non-experts using the above approach seems to be a viable spindle detection method in terms of both efficiency and accuracy. Our study examined the practical feasibility of applying this method, and it may provide a useful approach for sleep spindle detection.

## Supporting information

S1 Fig Generation of the non-expert group standard with definite spindles in stage N2. Left: Three-dimensional plot of the mean F1 score of individual non-experts at varying group thresholds (T-group) and overlap thresholds (T-overlap) when definite spindles are only considered. Top right corner: The mean F1 scores of non-experts at T-overlap = 0.30 with T-groups ranging from 0 to 0.95 at increments of 0.05. The highest average performance was at T-group = 0.35. The shading indicates the standard deviation. At T-group = 0.35 and T-overlap = 0.30, the mean F1 score shows a significant decreasing trend in the direction of the T-overlap axis. This level of the non-expert group consensus was the non-expert group standard with definite spindles (nEGS-1).
(TIF)

S2 Fig Generation of the non-expert group standard with indefinite spindles in stage N2. Left: Three-dimensional plot of the mean F1 score of individual non-experts at varying group thresholds (T-group) and overlap thresholds (T-overlap) when indefinite spindles are only considered. Top right corner: The mean F1 scores of non-experts at T-overlap = 0.30 with T-groups ranging from 0 to 0.95 at increments of 0.05. The highest average performance was at T-group = 0.05. The shading indicates the standard deviation. At T-group = 0.05 and T-overlap = 0.30, the mean F1 score shows a significant decreasing trend in the direction of the T-overlap axis. This level of the non-expert group consensus was the non-expert group standard with indefinite spindles (nEGS-05). The T-group ranges from 0 to 0.20 because the mean F1 score is not a number when the T-group ranges from 0.25 to 0.95.
(TIF)

S3 Fig Generation of the expert group standard in stage N3. Left: Three-dimensional plot of the mean F1 score of individual experts at varying group thresholds (T-group) and overlap thresholds (T-overlap). Top right corner: The mean F1 scores of experts at T-overlap = 0.40 with T-groups ranging from 0 to 0.95 at increments of 0.05. The highest average performance was at T-group = 0.30. The shading indicates the standard deviation. At T-group = 0.30 and T-overlap = 0.40, the mean F1 score shows a significant decreasing trend in the direction of the T-overlap axis, and the color clearly changes from dark red to orange (see left). This level of the expert group consensus was the expert group standard (EGS). Bottom right corner: A precision-recall plot of individual expert performance. Each square represents one expert. The square and the dot represent the performance with and without the leave-one-out method.
(TIF)

S4 Fig Generation of the non-expert group standard with all spindles in stage N3. Left: Three-dimensional plot of the mean F1 score of individual non-experts at varying group thresholds (T-group) and overlap thresholds (T-overlap) when both definite spindles and indefinite spindles are considered. Top right corner: The mean F1 scores of non-experts at T-overlap = 0.30 with T-groups ranging from 0 to 0.95 at increments of 0.05. The highest average performance was at T-group = 0.30. The shading indicates the standard deviation. At T-group = 0.30 and T-overlap = 0.30, the mean F1 score shows a significant decreasing trend in the direction of the T-overlap axis. This level of the non-expert group consensus was the non-expert group standard with all spindles (nEGS-all).
(TIF)

S5 Fig Generation of the non-expert group standard with definite spindles in stage N3. Left: Three-dimensional plot of the mean F1 score of individual non-experts at varying group thresholds (T-group) and overlap thresholds (T-overlap) when definite spindles are only considered. Top right corner: The mean F1 scores of non-experts at T-overlap = 0.25 with T-groups ranging from 0 to 0.95 at increments of 0.05. The highest average performance was at T-group = 0.30. The shading indicates the standard deviation. At T-group = 0.30 and T-overlap = 0.25, the mean F1 score shows a significant decreasing trend in the direction of the T-overlap axis. This level of the non-expert group consensus was the non-expert group standard with definite spindles (nEGS-1).
(TIF)

S6 Fig Generation of the non-expert group standard with indefinite spindles in stage N3. Left: Three-dimensional plot of the mean F1 score of individual non-experts at varying group thresholds (T-group) and overlap thresholds (T-overlap) when indefinite spindles are only considered. Top right corner: The mean F1 scores of non-experts at T-overlap = 0.35 with T-groups ranging from 0 to 0.95 at increments of 0.05. The highest average performance was at T-group = 0.05. The shading indicates the standard deviation. At T-group = 0.05 and T-overlap = 0.35, the mean F1 score shows a significant decreasing trend in the direction of the T-overlap axis. This level of the non-expert group consensus was the non-expert group standard with indefinite spindles (nEGS-05). The T-group ranges from 0 to 0.15 because the mean F1 score is not a number when the T-group ranges from 0.20 to 0.95.
(TIF)

S7 FigThe $\bar{\text{F1 score}}$ of EGS and nEGS-1, as well as the VS-F1-score of automatic methods and nEGS-1 versus EGS at different overlap thresholds in stages N2 (A) and N3 (B).The EGS is the expert group standard. The nEGS-1 is the non-expert group standard when definite spindles are only considered. The AS1, AS2, AS3 and AS4 are the automatic standards obtained by the first, second, third and fourth automated method, respectively. The $\bar{\text{F1 score}}$ is the mean F1 score of EGS and nEGS-1 at optimal thresholds. The VS-F1-score is the F1 score of nEGS-1, AS1, AS2, AS3 and AS4 compared with EGS using the matching procedure at an overlap threshold. The nEGS-1/EGS denotes a comparison of nEGS-1 with EGS.(TIF)

S8 FigCorrelation between the $\bar{\text{F1-score-each}}$ of EGS-each and the ${\text{mean}}_{500}\bar{\text{F1-score-each}}\text{(}6\text{)}$ of nEGS-1-6-each (A), and the mean_500_<VS-F1-score-each(6)> of nEGS-1-6-each compared with EGS-each in stage N2 sleep data (B).Note that after remove three outliers, the $\bar{\text{F1-score-each}}$ of EGS-each was still significant correlated with the ${\text{mean}}_{\text{500}}\bar{\text{F1-score-each}}\text{(6)}$ of nEGS-1-6-each (r = 0.61, P < 10–3). After remove two outliers, the $\bar{\text{F1-score-each}}$ of EGS-each was still significant correlated with the mean_500_<VS-F1-score-each(6)> of nEGS-1-6-each (r = 0.58, P < 0.001). The EGS-each is the expert group standard of each data segment. The nEGS-1-6-each is the non-expert group standard with definite spindles of each stage N2 sleep data segment from six non-experts. The $\bar{\text{F1-score-each}}$ was the mean F1 score of EGS-each of each data segment at optimal thresholds. The ${\text{mean}}_{\text{500}}<\bar{\text{F1-score-each}}(6)>$ is the mean F1 score of nEGS-1-6-each of each stage N2 data segment at the optimal thresholds across 500 repetitions. The mean_500_<VS-F1-score-each(6)> is the mean F1 score of nEGS-1-6-each compared with EGS-each across 500 repetitions.(TIF)

S9 Fig Three-dimensional plots of the mean F1 score of nEGS-1-6 in stage N2 (A) and that of nEGS-1-9 in stage N3 (B) across 500 repetitions at varying group thresholds and overlap thresholds. The nEGS-1-6 is the non-expert group standard with definite spindles from six non-experts identifying spindles in one data segment of stage N2. The nEGS-1-9 is the non-expert group standard with definite spindles from nine non-experts identifying spindles in one data segment of stage N3.
(TIF)

S1 File The methods and results in supporting information. (DOCX)

S2 File The relevant data within this study. This MAT-file includes two structs: N2 and N3, representing stage N2 and N3. Each struct contains four structs: EGS, nEGS, nEGS-1 and nEGS-05. The EGS struct is composed of the F1_score (a 20×20×5 matrix, the performance of five experts at varying T-groups and T-overlaps) and the standard (a 300,000×30 matrix, the spindle detection results of 30 data segments by experts group). Similarly, the nEGS, nEGS-1 and nEGS-05 structs consist of the F1_score (a 20×20×168 matrix, the performance of 168 non-experts at varying T-groups and T-overlaps) and the standard (a 300,000×30 matrix, the spindle detection results of 30 data segments by non-experts group). These three structs are generated when both definite and indefinite spindles, only considering definite spindles and indefinite spindles are considered, respectively.
(MAT)

S1 Table Parameters of four group standards of each data segment in stage N2 and N3 sleep data. (DOCX)

S2 Table The performance of non-expert group standards of each data segment compared against the expert group standard of each data segment in stage N2 and N3 sleep data. (DOCX)

S3 Table Pairwise comparisons of performance between experts in stage N2 and N3 sleep data. (DOCX)

S4 Table Definitions of some terms in this study. (DOCX)

S5 Table The results of the classification using the k-means clustering method. (DOCX)

S6 Table The performance of experts with different number. (DOCX)
