# Supplementary figures and images for: Sleep spindle detection based on non-experts: A validation study
Source: PLoS One. 2017 May 11;12(5):e0177437. doi: 10.1371/journal.pone.0177437 (PMC5426701; doi:10.1371/journal.pone.0177437)

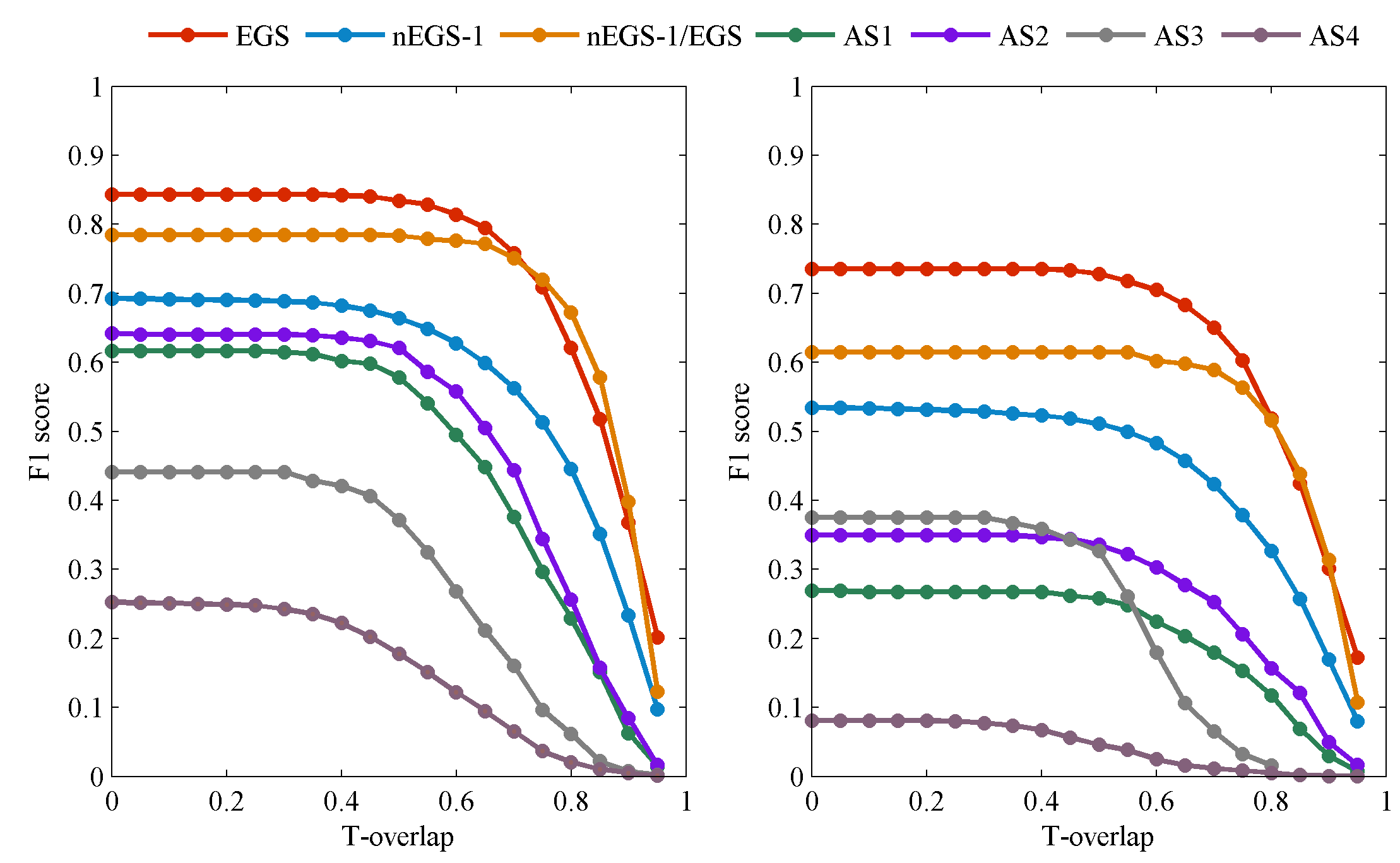

Supplement: S7 Fig — The EGS is the expert group standard. The nEGS-1 is the non-expert group standard when definite spindles are only considered. The AS1, AS2, AS3 and AS4 are the automatic standards obtained by the first, second, third and fourth automated method, respectively. The F1 score¯ is the mean F1 score of EGS and nEGS-1 at optimal thresholds. The VS-F1-score is the F1 score of nEGS-1, AS1, AS2, AS3 and AS4 compared with EGS using the matching procedure at an overlap threshold. The nEGS-1/EGS denotes a comparison of nEGS-1 with EGS. (TIF) [file pone.0177437.s007.tif]

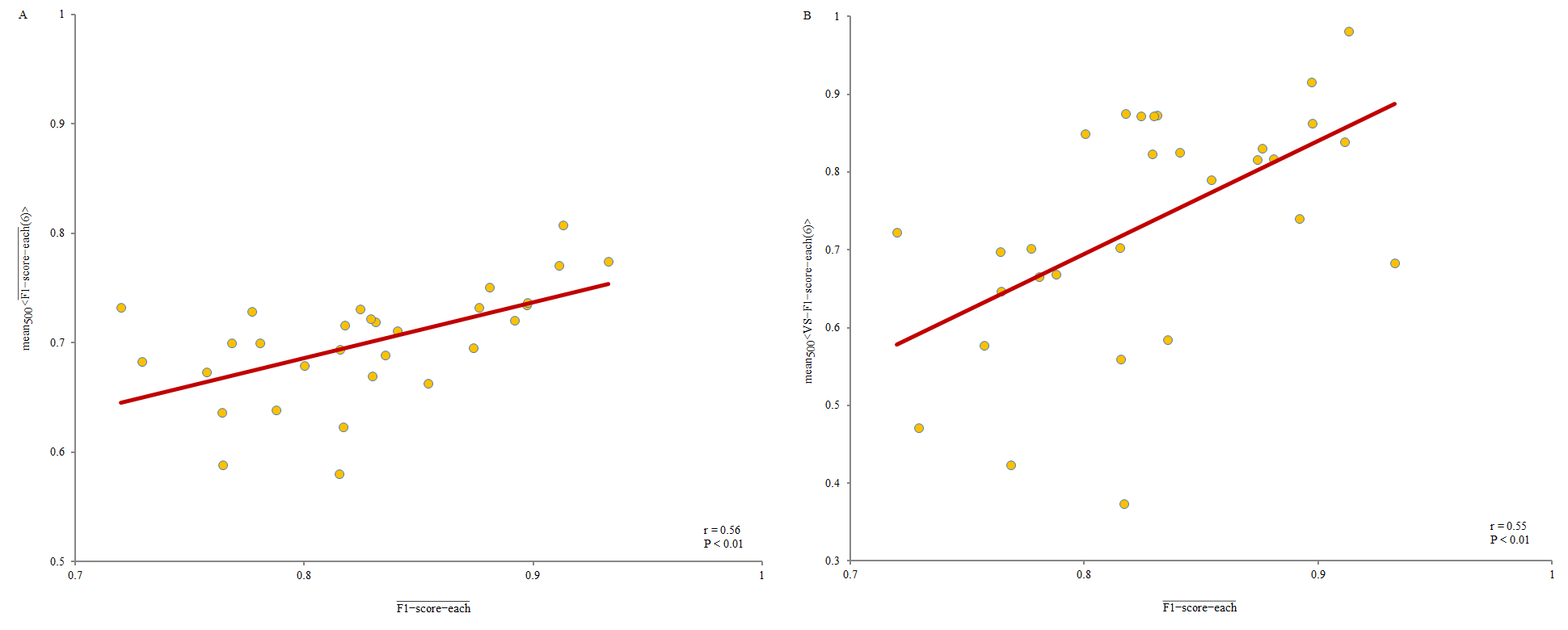

Supplement: S8 Fig — Note that after remove three outliers, the F1-score-each¯ of EGS-each was still significant correlated with the mean500F1-score-each¯(6) of nEGS-1-6-each (r = 0.61, P < 10–3). After remove two outliers, the F1-score-each¯ of EGS-each was still significant correlated with the mean500<VS-F1-score-each(6)> of nEGS-1-6-each (r = 0.58, P < 0.001). The EGS-each is the expert group standard of each data segment. The nEGS-1-6-each is the non-expert group standard with definite spindles of each stage N2 sleep data segment from six non-experts. The F1-score-each¯ was the mean F1 score of EGS-each of each data segment at optimal thresholds. The mean500<F1-score-each¯(6)> is the mean F1 score of nEGS-1-6-each of each stage N2 data segment at the optimal thresholds across 500 repetitions. The mean500<VS-F1-score-each(6)> is the mean F1 score of nEGS-1-6-each compared with EGS-each across 500 repetitions. (TIF) [file pone.0177437.s008.tif]
