# Supplementary material for: Sleep spindle detection based on non-experts: A validation study
Source: PLoS One. 2017 May 11;12(5):e0177437. doi: 10.1371/journal.pone.0177437 (PMC5426701; doi:10.1371/journal.pone.0177437)
Supplement: S1 File — (DOCX) [file pone.0177437.s010.docx]

**Supporting Information**

**Methods**

**Matching procedure**

The matching procedure was used to classifying two spindles into a classic 2 × 2 contingency table of true positive (TP), false positive (FP) and false negative (FN). We took the individual expert vector (also denoted as comparison vector), which was compared with the expert group vector (also called standard vector) at an overlap threshold (T-overlap), for example, to provide a brief outline.

Specifically, first, for the *i*th (*i* = 1 : I) spindle in the standard vector and the *j*th (*j* = 1 : J) spindle in the comparison vector, the intersection/union score ($\text{O}_{\text{ED}}\text{(}\text{i,j}\text{)}$) was calculated. E and D were the set of all of the spindles in the standard vector and in the comparison vector, respectively. I was the total number of spindles, and i was the *i*th spindle in the standard vector. J and j were the total number of spindles and the *j*th spindle in the comparison vector.

$\text{O}_{\text{ED}}\text{(}\text{i,j}\text{) = }\frac{\text{E(}\text{i}\text{)}\text{ }\text{∩}\text{ }\text{D(j)}}{\text{E(i)}\text{ }\text{∪}\text{ }\text{D(j)}}$ (1)

E(i) was the *i*th spindle in the standard vector. D(j) was the *j*th spindle in the comparison vector.

Second, we took the T-overlap on $\text{O}_{\text{ED}}\text{(}\text{i,j}\text{)}$ to classify D(j) and E(i) into the contingency table of TP, FP and FN, and converted $\text{O}_{\text{ED}}$ to a binary matrix (named Matching-matrix). The Matching-matrix(i,j) was 1 if $\text{O}_{\text{ED}}\text{(}\text{i,j}\text{)}$ exceeded T-overlap; otherwise, it was 0.

Third, we observed whether there was the 1 element in the *i*th row of Matching-matrix. If the *j_index*th element of the *i*th row of Matching-matrix was 1, the D(j_index) was the same as E(i). To ensure that only one D was the same as one E, we also observed whether the *i*th element of the *j_index*th column of Matching-matrix was 1. If the above condition was met, D(j_index) and E(i) were TP spindles. If all of the elements of the *i*th row of Matching-matrix were 0, the E(i) was FN spindle. If all of the elements of the *j*th column of Matching-matrix were 0, the D(j) was FP spindle.

However, several special situations must be considered. For example, when the D(j_index) was the same as E(i), the E(k) was the same as D(j_index) such that the *k*th element of the *j_index*th column of Matching-matrix was 1. Two spindles of E were the same as D(j_index), which did not meet the criterion that only one D is the same as one E. A similar situation would arise in which more than one spindle of D were the same as one E. To manage these situations, we again performed the above three steps for these paradoxical spindles only. A new intersection/union score ($\text{O}_{\text{ED}}\text{-}\text{new(}\text{i,j}\text{)}$, i = 1 : I_new, j = 1 : J_new) was calculated, and Matching-matrix-new(i,j) was obtained at T-overlap. I_new and J_new were the total number of paradoxical spindles in the standard vector and in the comparison vector, respectively. Then, these paradoxical spindles were classified into TP, FP or FN according to the third step. For further details, see Warby et al. [[1](#_ENREF_1)].

To summarize, the matching procedure was used to classify each spindle of the standard vector and the comparison vector into the contingency table of TP, FP and FN, which were determined by the intersection/union score. If the intersection/union score exceeded the overlap threshold (T-overlap), this spindle was counted as TP; otherwise, the spindle in the standard vector was counted as FN, and the spindle in the comparison vector was counted as FP. One criterion must meet that each spindle could only be classified once.

**Generation of the three non-expert group standards**

Three non-expert group standards (the non-expert group standard with all spindles, the non-expert group standard with definite spindles and the non-expert group standard with indefinite spindles) were established from 168 non-experts using the similar procedure as the expert group standard (Fig. 1B). We took the non-expert group standard with all spindles (nEGS-all), for example, to provide a brief outline. First, for the each data segment (n = 30), non-expert group consensus was obtained by averaging the identifications from multiple non-experts who identified this data segment. The non-expert group consensus and each non-expert score were applied at a group threshold (T-group, which increased from 0 to 0.95 in increments of 0.05), and respectively converted to non-expert group vector and individual non-expert vector. Using the matching procedure, individual non-expert vector was compared with the non-expert group vector at varying overlap thresholds (T-overlap = 0:0.05:0.95), and a classic contingency table of TP, FP and FN was established.

Second, for each non-expert (n = 168), the TP, FP and FN were added together across the data segments identified by this non-expert. Then, the F1 score (20 × 20 × 168 cases) were calculated at varying T-groups and T-overlaps. The mean F1 score was calculated by averaging the F1 scores of 168 non-experts. A 20 by 20 matrix of mean F1 scores was obtained at varying T-groups and T-overlaps, which was shown in a three-dimensional surface (X-axis: T-group, Y-axis: T-overlap, Z-axis: mean F1 score).

Finally, the optimal T-group was determined from the 3D surface by maximizing the mean F1 score of all individuals in the non-expert group, and the nEGS-all was established from the non-expert group consensus at the optimal T-group. The best T-overlap was selected by finding the first point at which the difference of the two adjacent values of the mean F1 score at the best T-group was more than 0.002.

For the non-expert group standard with definite spindles (nEGS-1), the weighted value of 1 of each non-expert score was only considered into the analysis. For non-expert group standard with indefinite spindles (nEGS-05), the weighted value of 0.5 of each non-expert score was only considered into the analysis. The generation procedure of these non-expert group standards was the same as nEGS-all.

**The comparison of the three non-expert group standards with EGS**

We compared the three non-expert group standards (nEGS-all, nEGS-1 and nEGS-05) with EGS at their respective best overlap thresholds using the matching procedure. For each data segment (n = 30), these non-expert group standards were compared with EGS at their respective T-overlaps, and the values of TP, FP and FN were obtained. For each non-expert group standard, the values of TP, FP and FN were added across 30 segments of data, and the recall, precision and F1 score (termed the VS-F1-score) were calculated.

**Generation of group standard of each data segment**

The procedure for generating the expert group standard of each data segment (EGS-each) was similar to that for the expert group standard (EGS). For each data segment (n = 30), we obtained the F1 score (20 × 20 × 5 cases) of the individual expert vector compared with the expert group vector at varying T-groups and T-overlaps using matching procedure. The mean F1 score was calculated by averaging the F1 scores of five experts. Then, the optimal thresholds were determined from the three-dimensional surface. The best T-group was selected by optimizing the mean F1 score of individuals in the expert group relative to its own group. The best T-overlap was the first point at which the difference of two adjacent values of the mean F1 score at the best T-group was more than 0.002. The mean F1 score of this point was termed $\bar{\text{F1-score-each}}$. The expert group consensus at the best T-group was the final EGS-each of this data segment. Therefore, we obtained 30 T-overlaps, 30 T-groups and 30 $\bar{\text{F1-score-each}}$.

In parallel, we generated non-expert group standard with definite spindles of each data segment (nEGS-1-each) from 168 non-experts. For each data segment (n = 30), we only considered the weight of 1 of individual non-expert scores into the analysis and obtained the best T-group, T-overlap and $\bar{\text{F1-score-each}}$.

For convenience, the means of the parameters of EGS-each and nEGS-1-each across 30 data segments were termed the $\text{mean}_{\text{30}}\text{T-grou}\text{p}$, $\text{mean}_{\text{30}}\text{T-}\text{overlap}$ and $\text{mean}_{\text{30}}\bar{\text{F1-score-each}}$.

**The comparison of nEGS-1-each with EGS-each**

For each data segment (n = 30), we compared nEGS-1-each with EGS-each at the best T-overlap using the matching procedure, and obtained the TP, FP, FN, recall, precision and VS-F1-score-each. We denoted the means of these parameters across 30 data segments as the $\text{mean}_{\text{30}}\text{T}\text{P}$, $\text{mean}_{\text{30}}\text{FP}$, $\text{mean}_{\text{30}}\text{FN}$, $\text{mean}_{\text{30}}\text{Recall}$, $\text{mean}_{\text{30}}\text{P}\text{recision}$ and $\text{mean}_{\text{30}}\text{VS-F1-score-each}$.

**The leave-one-out method for group standards**

The individual experts were compared to the expert group consensus to which their scores belonged, which gave them an advantage in the performance evaluation. To control for this, we evaluated the performance of individual experts using a leave-one-out method. Specifically, for each expert (n = 5), we established an expert group standard from the other four experts, and obtained the best T-group and T-overlap. Then, the F1 score was calculated by comparing this expert scores with the above expert group standard at T-group and T-overlap. The mean F1 score of five experts was calculated and termed the $\bar{\text{F1 score-leave}}$.

In parallel, we also evaluated the performance of individual non-experts using the leave-one-out method. Finally, the $\bar{\text{F1 score-leave}}$ values of 168 non-experts versus nEGS-all, nEGS-1 and nEGS-05 were obtained.

**The comparison of EGS-each with EGS**

We compared EGS-each with EGS at their respective best overlap thresholds using the matching procedure. For each data segment (n = 30), EGS-each was compared with EGS at the best T-overlap, and the values of TP, FP and FN were obtained. Then, the values of TP, FP and FN were added across 30 segments of data, and the recall, precision and F1 score (termed the VS-F1-score) were calculated.

**The comparison of nEGS-1-each with nEGS-1**

We compared nEGS-1-each with nEGS-1 at their respective best overlap thresholds using the matching procedure. For each data segment (n = 30), nEGS-1-each was compared with nEGS-1 at the best T-overlap, and the values of TP, FP and FN were obtained. Then, the values of TP, FP and FN were added across 30 segments of data, and the recall, precision and F1 score (termed the VS-F1-score-nEGS-1) were calculated.

**Pairwise comparisons of performance between experts**

We performed pairwise comparisons between any two experts at the best T-group and T-overlap of EGS using the matching procedure, and we obtained the F1 score (termed the pair-F1-score) for each pair. In total, 10 pair-F1-scores of five experts were calculated for both N2 and N3 sleep data.

**Generation of the non-expert group standard dependent of expert**

For each data segment (n = 30), the non-expert group consensus was calculated and converted to a binary vector by applying a T-group (T-group = 0:0.05:0.95). Then the binary vector was compared with the two-value vector of EGS at a T-overlap (T-overlap = 0:0.05:0.95) using the matching procedure, and an F1 score matrix (20 × 20) was obtained at varying T-groups and T-overlaps. Then, we determined the best T-group and T-overlap in the three-dimensional surface (X-axis: T-group, Y-axis: T-overlap, Z-axis: F1 score). The best T-group was selected by maximal the F1 score of non-expert group consensus compared to EGS. The best T-overlap was the first point at which the difference of two adjacent values of the F1 score at the best T-group was more than 0.002. The non-expert group consensus at the best T-group was the non-expert group standard dependent of expert, and the maximal F1 score (also termed VS-F1-score) was the performance of the non-expert group standard dependent of expert compared with EGS.

**Generation of nEGS-1-6 and nEGS-1-9**

The procedure used to generate the nEGS-1-6 (non-expert group standard with definite spindles from six non-experts identifying spindles in one data segment of stage N2) and the nEGS-1-9 (non-expert group standard with definite spindles from nine non-experts identifying spindles in one data segment of stage N3) was similar to that for nEGS-1. The difference was that the procedure was repeated 500 times with different non-experts.

Specifically, for each repetition, only the weighted value of 1 of non-expert scores was considered into the analysis. For each data segment (n = 30) of stage N2, the non-expert group consensus was calculated by averaging the identifications of six non-experts who identified this data segment. Each non-expert score was compared with the non-expert group consensus at varying T-groups (T-group = 0:0.05:0.95) and T-overlaps (T-overlap = 0:0.05:0.95) using the matching procedure, and the TP, FP and FN were established. Then, the TP, FP and FN of each non-expert were added together across 30 segments of data, and the F1 score was calculated. Then, the mean F1 scores corresponding to varying T-groups and T-overlaps were calculated by averaging the F1 scores of all non-experts who identified these 30 segments of data. Finally, the optimal thresholds were determined from the 3D surface by maximizing the mean F1 score of all individuals in the non-expert group. The nEGS-1-6 was established from the non-expert group consensus at the optimal T-group. Similarly, we also established the nEGS-1-9 from stage N3 data using the same procedure as nEGS-1-6.

For simplicity, we denoted the $\bar{\text{F1 score}}$ of nEGS-1-6 as $\bar{\text{F1 score}}\text{(6,i)}$, *i* = 1,2,3,…,500, and the $\bar{\text{F1 score}}$ of nEGS-1-9 as $\bar{\text{F1 score}}\text{(}\text{9}\text{,i)}$, *i* = 1,2,3,…,500 for each repetition.

**Generation of nEGS-1-6-each and nEGS-1-9-each**

The procedure for generating nEGS-1-6-each (the non-expert group standard with definite spindles of each data segment from six non-experts identifying spindles in one data segment of stage N2) and nEGS-1-9-each (the non-expert group standard with definite spindles of each data segment from nine non-experts identifying spindles in one data segment of stage N3) was similar to that for nEGS-1-each. The difference was that for each data segment the procedure was repeated 500 times. For each data segment (n = 30), we finally obtained 500 best T-groups, 500 T-overlaps and 500 $\bar{\text{F1}\text{-}\text{score}\text{-}\text{eac}\text{h}}$ values. The means of the parameters of nEGS-1-6-each and nEGS-1-9-each (termed the $\text{mean}_{\text{500}}\text{T-group}$, $\text{mean}_{\text{500}}\text{T-overlap}$, and $\text{mean}_{\text{500}}\text{<}\bar{\text{F1-score-each}}\text{>}$) were calculated across 500 repeats.

In consideration of clear expression, the mean parameters of nEGS-1-6-each and nEGS-1-9-each across 30 data segments (termed the $\text{mean}_{\text{30}}\text{<}\text{mean}_{\text{500}}\text{T-group}\text{>}$, $\text{mean}_{\text{30}}\text{<}\text{mean}_{\text{500}}\text{T-overlap}\text{>}$, and $\text{mean}_{\text{30}}\text{<}\text{mean}_{\text{500}}\text{<}\bar{\text{F1-score-each}}\text{>>}$) were collectively called the $\text{mean}_{\text{30}}\text{T-grou}\text{p}$, $\text{mean}_{\text{30}}\text{T-}\text{overlap}$, and $\text{mean}_{\text{30}}\bar{\text{F1-score-each}}$ (S1 Table).

**The comparison of nEGS-1-6-each and nEGS-1-9-each with EGS-each**

For each data segment (n = 30), we compared nEGS-1-each with EGS-each at the best T-overlap using the matching procedure, and obtained the TP, FP, FN, recall, precision and VS-F1-score-each. The best T-overlap corresponded to the nEGS-1-each of this data segment. We denoted the means of these parameters across 30 data segments as the $\text{mean}_{\text{30}}\text{T}\text{P}$, $\text{mean}_{\text{30}}\text{FP}$, $\text{mean}_{\text{30}}\text{FN}$, $\text{mean}_{\text{30}}\text{Recall}$, $\text{mean}_{\text{30}}\text{P}\text{recision}$ and $\text{mean}_{\text{30}}\text{VS-F1-score-each}$.

Similarly, for each data segment (n = 30), we performed 500 repeats to compare nEGS-1-6-each with EGS-each of N2 sleep data at the best T-overlap that corresponded to nEGS-1-6-each of this data segment. Finally, 500 TP, FP, FN, recall, precision and VS-F1-score-each values were obtained for each data segment. The means of these parameters (termed the $\text{mean}_{\text{500}}\text{TP}$, $\text{mean}_{\text{500}}\text{FP}$, $\text{mean}_{\text{500}}\text{FN}$, $\text{mean}_{\text{500}}\text{Recall}$,$\text{mean}_{\text{500}}\text{Precision}$ and $\text{mean}_{\text{500}}\text{<VS-F1-score-each>}$) with EGS-each were calculated across 500 repeats.

In parallel, for each data segment of stage N3 (n = 30), we compared nEGS-1-9-each with EGS-each 500 times at the best T-overlap that corresponded to nEGS-1-9-each of the data segment, and we obtained the above parameters.

In consideration of clear expression, the mean parameters of nEGS-1-6-each and nEGS-1-9-each versus EGS-each across 30 data segments ($\text{mean}_{\text{30}}\text{<}\text{mean}_{\text{500}}\text{TP>}$, $\text{mean}_{\text{30}}\text{<}\text{mean}_{\text{500}}\text{FP}\text{>}$, $\text{mean}_{\text{30}}\text{<}\text{mean}_{\text{500}}\text{FN}\text{>}$, $\text{mean}_{\text{30}}\text{<}\text{mean}_{\text{500}}\text{Recall}\text{>}$, $\text{mean}_{\text{30}}\text{<}\text{mean}_{\text{500}}\text{Precision}\text{>}$ and $\text{mean}_{\text{30}}\text{<}\text{mean}_{\text{500}}\text{<VS-F1-score-each>>}$) were collectively called $\text{mean}_{\text{30}}\text{TP}$ $\text{mean}_{\text{30}}\text{FP}$, $\text{mean}_{\text{30}}\text{FN}$, $\text{mean}_{\text{30}}\text{Recall}$, $\text{mean}_{\text{30}}\text{P}\text{recision}$ and $\text{mean}_{\text{30}}\text{VS-F1-score-each}$, respectively (S2 Table).

**Results**

**The performance of the non-expert group standard dependent of expert**

The VS-F1-scores of the non-expert group standard dependent of expert were 0.84 and 0.79 for stage N2 and N3, respectively. The VS-F1-score of the non-expert group standard dependent of expert in stage N2 was higher than that of Warby et al. (F1 score = 0.67), which may have resulted from the EEG data set being extracted from young subjects in the present study. The spindle amplitudes of young subjects are relatively large, and the shape is stable. However, in middle- and older-aged subjects, spindle amplitude and duration are on the decline [[2](#_ENREF_2), [3](#_ENREF_3)], which impairs the performance of scorers manually identifying spindles.

**The performance of nEGS-1-6 and nEGS-1-9**

We established nEGS-1-6 (the non-expert group standard with definite spindles from six non-experts identifying spindles in one data segment of stage N2, see details in methods of Supporting information) when there were six non-experts identifying spindles in one stage N2 sleep data segment. The $\bar{\text{F1 score}}\text{(}\text{6}\text{,i)}$ (*i* = 1,2,3,…,500) of nEGS-1-6 ranged from 0.68 to 0.73, and the VS-F1-score(6,i) (*i* = 1,2,3,…,500) of nEGS-1-6 versus EGS ranged from 0.72 to 0.80. A three-dimensional plot of the mean F1 scores of nEGS-1-6 at varying T-groups and T-overlaps across 500 repeats is shown in S9A Fig. In parallel, for stage N3, we also generated nEGS-1-9 (the non-expert group standard with definite spindles from nine non-experts identifying spindles in one data segment of stage N3) when there were nine non-experts identifying spindles in one stage N3 sleep data segment (see methods in S1 File). The $\bar{\text{F1 score}}\text{(}\text{9}\text{,i)}$ (*i* = 1,2,3,…,500) of nEGS-1-9 ranged from 0.55 to 0.56, and the VS-F1-score(9,i) (*i* = 1,2,3,…,500) of nEGS-1-9 versus EGS ranged from 0.51 to 0.71. A three-dimensional plot of nEGS-1-9 across 500 repeats is presented in S9B Fig. These findings indicate that the performance of nEGS-1-6 or nEGS-1-9 across 500 repeats was relatively high and stable.

**The performance of nEGS-1-6-each and nEGS-1-9-each**

We also generated nEGS-1-6-each (the non-expert group standard with definite spindles of each data segment from six non-experts identifying spindles in one data segment of stage N2) and nEGS-1-9-each (the non-expert group standard with definite spindles of each data segment from nine non-experts identifying spindles in one data segment of stage N3) (see methods in S1 File). The mean parameters of nEGS-1-6-each or nEGS-1-9-each and the mean performance of nEGS-1-6-each or nEGS-1-9-each versus EGS-each across 30 data segments are shown in S1 and S2 Tables, respectively. For stage N2, Pearson’s correlation analysis demonstrated that the $\bar{\text{F1-score-each}}$ of EGS-each was not only related to the $\text{mean}_{\text{500}}\text{<}\bar{\text{F1-score-each}}\text{(6)>}$ of nEGS-1-6-each (r = 0.56, P < 0.01; S8A Fig) but was also correlated to the $\text{mean}_{\text{500}}\text{<VS-F1-score-each(6)>}$ of nEGS-1-6-each versus EGS-each (r = 0.55, P < 0.01; S8B Fig). We also found a correlation between the $\text{mean}_{\text{500}}\text{<}\bar{\text{F1-score-each}}\text{(6)>}$ and $\text{mean}_{\text{500}}\text{<VS-F1-score-each(6)>}$ of nEGS-1-6-each (r = 0.49, P < 0.01).

$\text{mean}_{\text{500}}\text{<}\bar{\text{F1-score-each}}\text{(6)> = }\frac{\text{1}}{\text{500}}\times\sum_{\text{i = 1}}^{\text{500}} \bar{\text{F1-score-each}}\text{(6,i)}$ (2)

$\bar{\text{F1-score-each}}\text{(6,i)}$ was the maximal mean F1 score of nEGS-1-6-each when repeated the *i*th time.

$\text{mean}_{\text{500}}\text{<VS-F1-score-each(6)> = }\frac{\text{1}}{\text{500}}\times\sum_{\text{i = 1}}^{\text{500}} \text{VS-F1-score-each(6,i)}$ (3)

The $\text{VS-F1-score-}\text{each(}\text{6,i)}$ was the F1 score of nEGS-1-6-each versus EGS-each when repeated the *i*th time.

However, a partial correlation analysis revealed relationships only between the $\bar{\text{F1-score-each}}$ of EGS-each and the $\text{mean}_{\text{500}}\text{<}\bar{\text{F1-score-each}}\text{(6)>}$ of nEGS-1-6-each (r = 0.40, P < 0.05; S8A Fig), as well as between the $\bar{\text{F1-score-each}}$ of EGS-each and the $\text{mean}_{\text{500}}\text{<VS-F1-score-each(6)>}$ of nEGS-1-6-each (r = 0.38, P < 0.05; S8B Fig). For stage N3, we found no correlations among these factors in Pearson’s correlation analysis or in partial correlation analysis. These results also support the hypothesis that the data with higher performance by experts had higher performance by non-experts, especially for stage N2.

**References**

1. Warby SC, Wendt SL, Welinder P, Munk EG, Carrillo O, Sorensen HB, et al. Sleep-spindle detection: crowdsourcing and evaluating performance of experts, non-experts and automated methods. Nature methods. 2014;11(4):385-92.

2. Nicolas A, Petit D, Rompre S, Montplaisir J. Sleep spindle characteristics in healthy subjects of different age groups. Clinical Neurophysiology. 2001;112(3):521-7.

3. Martin N, Lafortune M, Godbout J, Barakat M, Robillard R, Poirier G, et al. Topography of age-related changes in sleep spindles. Neurobiology of aging. 2013;34(2):468-76.
