# Supplementary material for: Sleep spindle detection based on non-experts: A validation study
Source: PLoS One. 2017 May 11;12(5):e0177437. doi: 10.1371/journal.pone.0177437 (PMC5426701; doi:10.1371/journal.pone.0177437)
Supplement: S1 Table — (DOCX) [file pone.0177437.s012.docx]

**S1 Table. Parameters of four group standards of each data segment in stage N2 and N3 sleep data.**

| **Stage** | **Standard** | $\text{mean}_{\text{30}}\text{T-group}$ | $\text{mean}_{\text{30}}\text{T-overlap}$ | $\text{mean}_{\text{30}}\bar{\text{F1-score-each}}$ |
| --- | --- | --- | --- | --- |
| **N2** | **EGS-each** | 0.34 ± 0.09 | 0.50 ± 0.08 | 0.83 ± 0.06 |
|  | **nEGS-1-each** | 0.40 ± 0.05 | 0.29 ± 0.09 | 0.67 ± 0.06 |
|  | **nEGS-1-6-each** | 0.34 ± 0.04 | 0.34 ± 0.12 | 0.70 ± 0.07 |
| **N3** | **EGS-each** | 0.38 ± 0.12 | 0.59 ± 0.09 | 0.78 ± 0.07 |
|  | **nEGS-1-each** | 0.34 ± 0.06 | 0.33 ± 0.14 | 0.54 ± 0.05 |
|  | **nEGS-1-9-each** | 0.32 ± 0.08 | 0.41 ± 0.15 | 0.58 ± 0..07 |

Data are presented as mean ± standard deviation. The EGS-each was the expert group standard of each data segment. The nEGS-1-each was the non-expert group standard with definite spindles of each data segment. The nEGS-1-6-each was the non-expert group standard with definite spindles of each data segment from six non-experts identifying spindles in one data segment of stage N2. The nEGS-1-9-each was the non-expert group standard with definite spindles of each data segment from nine non-experts identifying spindles in one data segment of stage N3. The $\text{mean}_{\text{30}}\text{T-group}$ is the mean of the best group thresholds across 30 data segments of each group standard. The $\text{mean}_{\text{30}}\text{T-overlap}$ is the mean of the best overlap thresholds across 30 data segments of each group standard. The $\text{mean}_{\text{30}}\bar{\text{F1-score-each}}$ is the mean of the mean F1 score of each group standard at optimal thresholds across 30 data segments
