# Supplementary material for: Sleep spindle detection based on non-experts: A validation study
Source: PLoS One. 2017 May 11;12(5):e0177437. doi: 10.1371/journal.pone.0177437 (PMC5426701; doi:10.1371/journal.pone.0177437)
Supplement: S2 Table — (DOCX) [file pone.0177437.s013.docx]

**S2 Table**. **The performance of non-expert group standards of each data segment compared against the expert group standard of each data segment in stage N2 and N3 sleep data.**

| **Stage** | **Standard** | $\text{mean}_{\text{30}}\text{TP}$ | $\text{mean}_{\text{30}}\text{FP}$ | $\text{mean}_{\text{30}}\text{FN}$ | $\text{mean}_{\text{30}}\text{Recall}$ | $\text{mean}_{\text{30}}\text{Precision}$ | $\text{mean}_{\text{30}}\text{VS-F1-score-each}$ |
| --- | --- | --- | --- | --- | --- | --- | --- |
| **N2** | **nEGS-1-each** | 17.03 ± 8.85 | 7.43 ± 5.04 | 1.00 ± 1.53 | 0.96 ± 0.06 | 0.67 ± 0.20 | 0.77 ± 0.15 |
|  | **nEGS-1-6-each** | 17.39 ± 8.89 | 9.84 ± 5.67 | 0.64 ± 1.21 | 0.97 ± 0.05 | 0.62 ± 0.19 | 0.74 ± 0.15 |
| **N3** | **nEGS-1-each** | 4.17 ± 4.06 | 4.23 ± 2.87 | 0.40 ± 0.56 | 0.84 ± 0.31 | 0.44 ± 0.22 | 0.63 ± 0.14 |
|  | **nEGS-1-9-each** | 4.23 ± 4.03 | 5.27 ± 3.89 | 0.33 ± 0.58 | 0.88 ± 0.27 | 0.43 ± 0.23 | 0.59 ± 0.18 |

Data are presented as mean ± standard deviation. The nEGS-1-each is the non-expert group standard with definite spindles of each data segment. The nEGS-1-6-each was the non-expert group standard with definite spindles of each stage N2 data segment from six non-experts. The nEGS-1-9-each was the non-expert group standard with definite spindles of each stage N3 data segment from nine non-experts. The $\text{mean}_{\text{30}}\text{TP}$, $\text{mean}_{\text{30}}\text{FP}$, $\text{mean}_{\text{30}}\text{FN}$, $\text{mean}_{\text{30}}\text{Recall}$, $\text{mean}_{\text{30}}\text{Precision}$ and $\text{mean}_{\text{30}}\text{VS-F1-score-each}$ are the mean of true positive, false positive, false negative, recall, precision and F1 score of standards compared against EGS-each across 30 data segments, respectively.
