# Supplementary material for: Sleep spindle detection based on non-experts: A validation study
Source: PLoS One. 2017 May 11;12(5):e0177437. doi: 10.1371/journal.pone.0177437 (PMC5426701; doi:10.1371/journal.pone.0177437)
Supplement: S3 Table — (DOCX) [file pone.0177437.s014.docx]

**S3 Table. Pairwise comparisons of performance between experts in stage N2 and N3 sleep data.**

| **Stage** | **Pair-experts** | **TP** | **FP** | **FN** | **Recall** | **Precision** | **Pair-F1-score** |
| --- | --- | --- | --- | --- | --- | --- | --- |
| **N2** | **e1-2** | 379 | 272 | 71 | 0.84 | 0.58 | 0.69 |
|  | **e1-3** | 424 | 227 | 143 | 0.75 | 0.65 | 0.70 |
|  | **e1-4** | 523 | 128 | 184 | 0.74 | 0.80 | 0.77 |
|  | **e1-5** | 442 | 209 | 137 | 0.76 | 0.68 | 0.72 |
|  | **e2-3** | 353 | 97 | 214 | 0.62 | 0.78 | 0.69 |
|  | **e2-4** | 429 | 21 | 278 | 0.61 | 0.95 | 0.74 |
|  | **e2-5** | 373 | 77 | 206 | 0.64 | 0.83 | 0.73 |
|  | **e3-4** | 496 | 71 | 211 | 0.70 | 0.87 | 0.78 |
|  | **e3-5** | 417 | 150 | 162 | 0.72 | 0.74 | 0.73 |
|  | **e4-5** | 488 | 219 | 91 | 0.84 | 0.69 | 0.76 |
| **N3** | **e1-2** | 94 | 127 | 25 | 0.79 | 0.43 | 0.55 |
|  | **e1-3** | 120 | 101 | 32 | 0.79 | 0.54 | 0.64 |
|  | **e1-4** | 190 | 31 | 164 | 0.54 | 0.86 | 0.66 |
|  | **e1-5** | 98 | 123 | 33 | 0.75 | 0.44 | 0.56 |
|  | **e2-3** | 83 | 36 | 69 | 0.55 | 0.70 | 0.61 |
|  | **e2-4** | 114 | 5 | 240 | 0.32 | 0.96 | 0.48 |
|  | **e2-5** | 85 | 34 | 46 | 0.65 | 0.71 | 0.68 |
|  | **e3-4** | 133 | 19 | 221 | 0.38 | 0.88 | 0.53 |
|  | **e3-5** | 85 | 67 | 46 | 0.65 | 0.56 | 0.60 |
|  | **e4-5** | 119 | 235 | 12 | 0.91 | 0.34 | 0.49 |

Pair-experts are two experts compared with each other. The TP is true positive. The FP is false positive. The FN is false negative. The Recall is equal to TP divided by the sum of TP and FN. The Precision is equal to TP divided by the sum of TP and FP. The Pair-F1-score is the F1 score of two experts compared with each other.
