# Supplementary material for: Sleep spindle detection based on non-experts: A validation study
Source: PLoS One. 2017 May 11;12(5):e0177437. doi: 10.1371/journal.pone.0177437 (PMC5426701; doi:10.1371/journal.pone.0177437)
Supplement: S4 Table — (DOCX) [file pone.0177437.s015.docx]

**S4 Table. Definitions of some terms in this study.**

| **Terms** | **Definitions** |
| --- | --- |
| EGS | = the expert group standard |
| expert/non-expert group  consensus | = the averaged vector of the expert/non-expert group |
| expert/non-expert group  vector | = the expert/non-expert group consensus with a T-group applied and converted to a binary vector |
| individual expert/non-expert  vector | = the individual expert/non-expert scores with a T-group applied and converted to a binary vector |
| expert/non-expert group  standard | = the expert/non-expert group consensus with an optimal T-group applied and converted to a binary vector |
| true positive (TP) | = correct detection |
| false positive (FP) | = incorrect detection |
| false negative (FN) | = incorrect non-detection |
| group threshold (T-group) | = threshold for the expert/non-expert group consensus |
| intersection/union score  ($\text{O}_{\text{ED}}$) | = overlap ratio of two spindles  $\text{O}_{\text{ED}}\text{ = }\frac{\text{E∩D}}{\text{E}\text{∪}\text{D}}$ |
| E | = individual spindle in the standard vector. |
| D | = individual spindle in the comparison vector |
| overlap threshold (T-overlap) | = the required amount of overlap between two spindles to determine a TP |
| recall | = fraction of true spindles found  $\text{recall = }\frac{\text{TP}}{\text{TP + FN}}$ |
| precision | = fraction of spindles that are correct  $\text{precision = }\frac{\text{TP}}{\text{TP + FP}}$ |
| F1 score | = the harmonic mean of precision and recall  $\text{F1 score = }\frac{\text{2 × recall × precision}}{\text{recall + precision}}$ |
| nEGS-all | = non-expert group standard with all spindles containing both the weights of 1 and 0.5 |
| nEGS-1 | = non-expert group standard with definite spindles only considering the weight of 1 |
| nEGS-05 | = non-expert group standard with indefinite spindles only considering the weight of 0.5 |
| group standard | = the general term for EGS, nEGS-all, nEGS-1 and nEGS-05 |
| AS1 | = the automatic standard obtained by the first automated method |
| AS2 | = the automatic standard obtained by the second automated method |
| AS3 | = the automatic standard obtained by the third automated method |
| AS4 | = the automatic standard obtained by the fourth automated method |
| $\bar{\text{F1 score}}$ | = the mean F1 score of group standard at optimal thresholds |
| VS-F1-score | = the F1 score of group standard or AS compared with EGS using the matching procedure |
| EGS-each | = the expert group standard of each data segment |
| nEGS-1-each | = the non-expert group standard with definite spindles of each data segment |
| $\bar{\text{F1-score-each}}$ | = the mean F1 score of the group standards (EGS-each and nEGS-1-each) of each data segment at optimal thresholds |
| VS-F1-score-each | = the F1 score of the nEGS-1-each versus EGS-each. |
| $\bar{\text{F1 score-leave}}$ | = the mean F1 score of five experts or 168 non-experts versus group standard in the leave-one-out analysis. |
| pair-F1-score | = the F1 score of two experts compared with each other. |
| VS-F1-score-nEGS-1 | = the F1 score of nEGS-1-each versus nEGS-1. |
| nEGS-1-6 | = the non-expert group standard with definite spindles from six non-experts identifying spindles in one data segment of stage N2 |
| nEGS-1-9 | = the non-expert group standard with definite spindles from nine non-experts identifying spindles in one data segment of stage N3 |
| nEGS-1-6-each | = the non-expert group standard with definite spindles of each data segment from six non-experts identifying spindles in one data segment of stage N2 |
| nEGS-1-9-each | = the non-expert group standard with definite spindles of each data segment from nine non-experts identifying spindles in one data segment of stage N3 |
