# Supplementary material for: Sleep spindle detection based on non-experts: A validation study
Source: PLoS One. 2017 May 11;12(5):e0177437. doi: 10.1371/journal.pone.0177437 (PMC5426701; doi:10.1371/journal.pone.0177437)
Supplement: S5 Table — (DOCX) [file pone.0177437.s016.docx]

**S5 Table. The results of the classification using the k-means clustering method.**

| **Non-experts** | **N2** | | | **N3** | | |
| --- | --- | --- | --- | --- | --- | --- |
|  | **F1 scores** | **Cluster 1** | **Cluster 2** | **F1 scores** | **Cluster 1** | **Cluster 2** |
| **1** | 21 | 21 | 0 | 21 | 21 | 0 |
| **2** | 210 | 210 | 0 | 210 | 210 | 0 |
| **3** | 500 | 426 | 74 | 500 | 245 | 255 |
| **4** | 500 | 417 | 83 | 500 | 363 | 137 |
| **5** | 500 | 362 | 138 | 500 | 466 | 34 |
| **6** | 500 | 12 | 488 | 500 | 114 | 386 |
| **7** | 500 | 73 | 427 | 500 | 51 | 449 |
| **8** | 500 | 100 | 400 | 500 | 201 | 299 |
| **9** | 500 | 0 | 500 | 500 | 130 | 370 |
| **10** | 500 | 1 | 499 | 500 | 9 | 491 |
| **11** | 500 | 4 | 496 | 500 | 15 | 485 |
| **12** | 500 | 0 | 500 | 500 | 89 | 411 |
| **13** | 500 | 0 | 500 | 500 | 29 | 471 |
| **14** | 500 | 0 | 500 | 500 | 3 | 497 |
| **15** | 500 | 0 | 500 | 500 | 6 | 494 |
| **16** | 500 | 0 | 500 | 500 | 22 | 478 |
| **17** | 500 | 0 | 500 | 500 | 7 | 493 |
| **18** | 500 | 0 | 500 | 500 | 1 | 189 |
| **19** | 210 | 0 | 210 | 210 | 0 | 210 |
| **20** | 21 | 0 | 21 | 21 | 0 | 21 |

The Non-expert was the number of non-experts identifying sleep spindles. The F1 scores was the total number of F1 scores in each condition. The Cluster 1 was the number of F1 scores in Cluster 1 with low performance. The Cluster 2 was the number of F1 scores in Cluster 2 with high performance.
