# Supplementary material for: Sleep spindle detection based on non-experts: A validation study
Source: PLoS One. 2017 May 11;12(5):e0177437. doi: 10.1371/journal.pone.0177437 (PMC5426701; doi:10.1371/journal.pone.0177437)
Supplement: S6 Table — (DOCX) [file pone.0177437.s017.docx]

**S6 Table. The performance of experts with different number.**

| **Experts** | **N2** | **N3** |
| --- | --- | --- |
| **1** | 0.84 ± 0.03 | 0.71 ± 0.06 |
| **2** | 0.85 ± 0.02 | 0.75 ± 0.08 |
| **3** | 0.92 ± 0.02 | 0.84 ± 0.03 |
| **4** | 0.94 ± 0.01 | 0.87 ± 0.01 |

Data are presented as mean ± standard deviation unless otherwise indicated. The Experts was the number of experts identifying spindles. The value was the mean performance of experts versus gold standard over 5 or 10 times.
